# The Sphingolipid Rheostat in Female Reproductive Pathologies: From Molecular Mechanisms to Precision Therapeutics

**DOI:** 10.1017/erm.2026.10070

**Published:** 2026-08-04

**Authors:** Ruolin Mao, Lei Jin, Wei Liu

**Affiliations:** Reproductive Medicine Center, Tongji Hospital, Tongji Medical College, Huazhong University of Science and Technology, Wuhan, China

**Keywords:** endometriosis, oocyte quality, pathological fibrosis, PCOS, precision medicine, sphingolipid rheostat

## Abstract

**Background:**

The sphingolipid rheostat – the interconversion between pro-apoptotic ceramide (Cer) and pro-survival sphingosine-1-phosphate (S1P) – governs female reproductive homeostasis. This review integrates mechanistic evidence across the reproductive continuum to elucidate how compartmentalised disruptions of this axis drive divergent pathologies.

**Methods:**

A systematic search up to 2026 across PubMed and Web of Science identified molecular mechanisms and translational models linking sphingolipid signalling to ovarian ageing, polycystic ovary syndrome (PCOS), endometriosis, adenomyosis and uterine fibroids.

**Results:**

Pathological Cer accumulation drives mitochondrial outer membrane permeabilisation (MOMP) and cytochrome c release, accelerating follicular atresia in primary ovarian insufficiency (POI). Conversely, PCOS is associated with heterogeneous remodeling of follicular-fluid sphingolipids, compromising oocyte competence. Within uterine and peritoneal microenvironments, a hyper-activated S1P axis operating via S1PR1–3 networks acts as a core pathogenic engine. This axis exhibits a threshold-dependent dual nature: low thresholds promote homeostatic survival, whereas high thresholds drive M2 macrophage polarisation for immune evasion and cross-link with TGF-β/Smad and Activin A cascades to stimulate fibroblast-to-myofibroblast transdifferentiation (FMT) and extracellular matrix deposition in endometriosis and fibroids. Additionally, the SphK/S1P axis amplifies chronic pelvic pain by modulating nerve growth factor (NGF) and transient receptor potential vanilloid 4 channels.

**Conclusions:**

The sphingolipid axis constitutes a unifying molecular rheostat in reproductive medicine. Mapping these compartmentalised lipidomic signatures enables high-resolution diagnostics, while targeted sub-receptor antagonists and non-hormonal drug repurposing offer promising, fertility-preserving therapeutic frontiers. However, successfully translating these lipid-targeted interventions into clinical practice demands addressing key bottlenecks regarding systemic off-target toxicities and engineering advanced, tissue-specific delivery platforms.

## Introduction

The dynamic homeostasis of the female reproductive system is maintained by a highly regulated sequence of cellular events, whose dysregulation manifests as a spectrum of severe molecular pathologies (Ref. 1). While the genomic and proteomic landscapes of reproductive disorders have been extensively mapped (Refs 2, 3), these static profiles often fail to capture the real-time metabolic fluxes that dictate cell fate. Consequently, the lipidome – specifically the bioactive sphingolipid network – has emerged as a critical determinant of cellular survival and organ-level functional integrity (Refs 4, 5). Sphingolipids are no longer characterised merely as structural constituents of biological membranes; instead, they are recognised as pivotal lipid secondary messengers that orchestrate key signal transduction nodes, directly cross-linking with the PI3K/Akt/mTOR, MAPK/ERK and TGF-β/Smad cascades (Ref. 6).

Central to this lipid signalling network is the sphingolipid rheostat, a metabolic pivot governed by the interconversion of pro-apoptotic ceramide (Cer) and pro-survival sphingosine-1-phosphate (S1P). This rheostat operates as a decisive molecular switch in reproductive tissues: physiological S1P gradients drive primordial follicle activation, stabilise the vascular endothelium and support oocyte developmental competence (Ref. 7). Conversely, a pathological shift towards ceramide accumulation facilitates mitochondrial outer membrane permeabilisation (MOMP) and cytochrome c release, driving the accelerated follicular atresia that underpins reproductive ageing and primary ovarian insufficiency (POI) (Ref. 8).

Current literature remains fragmented, with mechanistic research often compartmentalised into either ovarian physiology or isolated gynaecological malignancies (Ref. 8). While previous seminal reviews have discussed S1P dynamics in endometriosis or specific sphingolipid enzymes in folliculogenesis (Ref. 9), a multi-dimensional integration that connects these metabolic shifts across the entire reproductive tract is severely lacking. To bridge this gap, this review transcends these singular perspectives by systematically evaluating the sphingolipid axis across the female reproductive continuum – spanning from the ovary to the uterine and peritoneal microenvironments. We examine how a singular metabolic node can yield divergent clinical phenotypes depending on its spatial context: ranging from the accelerated depletion of the ovarian reserve in POI and metabolic silencing in polycystic ovary syndrome (PCOS) to the hyper-proliferative, fibrotic and nociceptive microenvironments of endometriosis, adenomyosis and uterine fibroids.

Furthermore, the clinical significance of sphingolipid signalling extends beyond disease pathogenesis into precision medicine. The distinctive sphingolipid metabolic signatures in follicular fluid (FF) and serum offer high-resolution molecular candidates for non-invasive diagnostics and the prediction of assisted reproductive outcomes (Ref. 10). Moreover, the successful application of S1P receptor modulators in systemic inflammatory conditions has opened innovative avenues for drug repurposing, providing non-hormonal therapeutic alternatives that treat lesions without inducing a hypoestrogenic state or disrupting a patient’s desire for conception. By delineating the biochemical crosstalk between sphingolipid metabolites and disease-specific signalling hubs across a diverse spectrum of disorders, this review provides a strategic blueprint for next-generation, lipid-targeted therapeutics in women’s reproductive medicine.

## Sources and selection criteria

This comprehensive review was conducted by performing a systematic and extensive literature search across the PubMed, Web of Science and Google Scholar databases for peer-reviewed articles published between 1996 and 2026. The search strategy employed an exhaustive combination of Boolean operators (AND/OR) and medical subject headings terms.

The core search strings integrated sphingolipid metabolic components (‘sphingolipids’, ‘sphingolipid rheostat’, ‘ceramide’, ‘sphingosine’, ‘sphingosine-1-phosphate’, and ‘sphingosine kinase’) with specific reproductive compartments and clinical entities. These entities included ovarian-centric conditions (‘oocyte quality’, ‘ovarian ageing’, ‘primary ovarian insufficiency’ and ‘polycystic ovary syndrome’), uterine and endometrial pathologies (‘endometriosis’, ‘adenomyosis’, ‘uterine fibroids’, ‘endometrial receptivity’, ‘pathological fibrosis’, ‘angiogenesis’ and ‘chronic pelvic pain’), as well as oncofertility paradigms (‘chemotherapy-induced gonadotoxicity’, ‘ovarian cancer’ and ‘fertility preservation’). Only articles published in English and focusing on high-resolution molecular mechanisms, translational animal models or human clinical trials were selected for inclusion in this review.

## The sphingolipid metabolic network and molecular features

### The sphingolipid rheostat: a decisive signalling hub

Sphingolipids are a diverse class of structural lipids defined by their 18-carbon amino alcohol sphingosine backbone (Ref. 11). Beyond serving as cell membrane scaffolds, they function as bioactive secondary messengers that orchestrate key physiological and pathological signal transduction nodes (Ref. 12). Within this metabolic network, three primary metabolites – ceramide, sphingosine and S1P – form a fundamental regulatory axis termed the ‘sphingolipid rheostat’ (Ref. 4).

**Figure 1.**
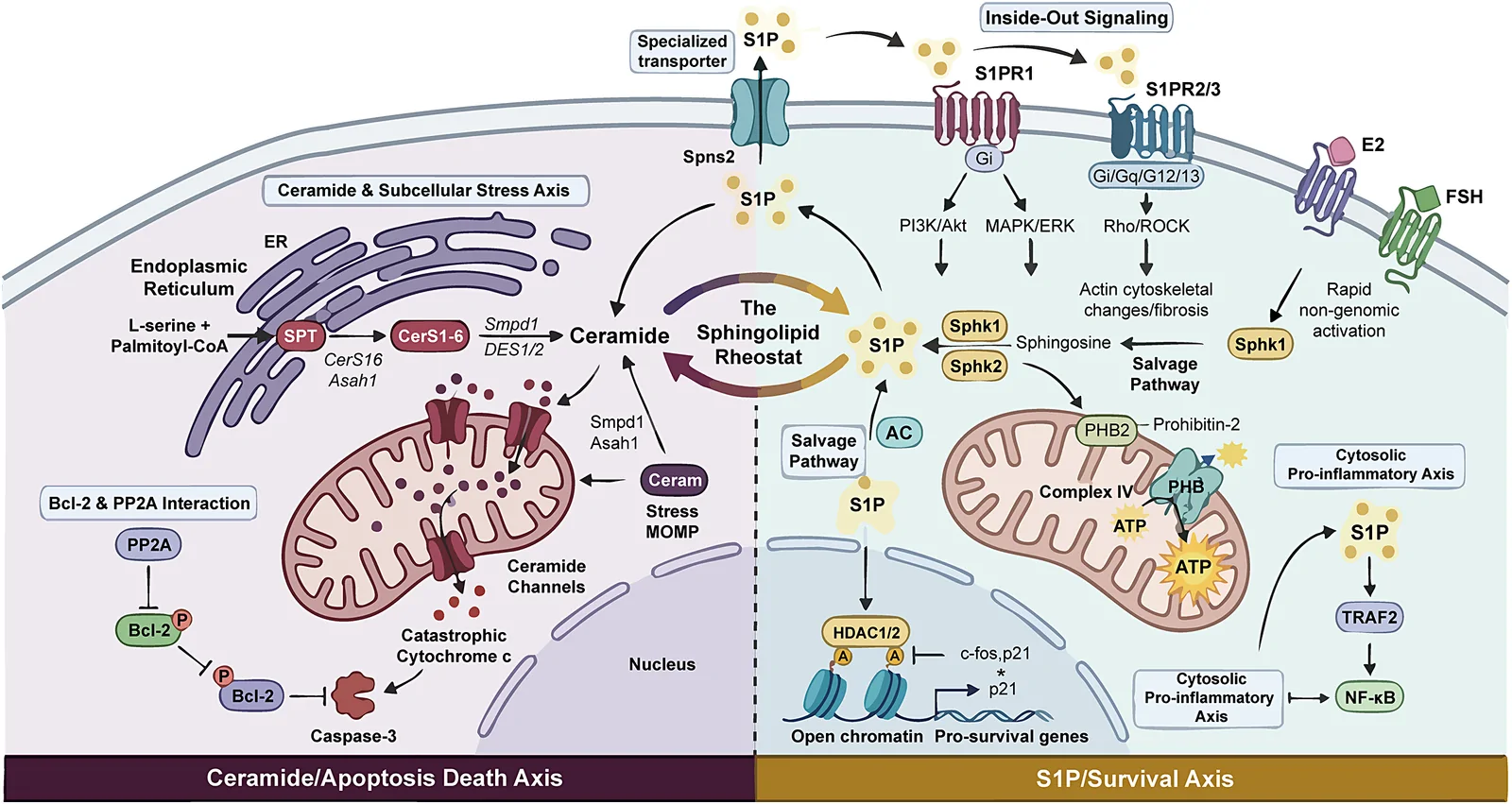
Subcellular topography of the sphingolipid rheostat: organelle crosstalk and signalling dynamics. The intracellular fate of reproductive cells is dictated by the dynamic balance between Cer and S1P across distinct subcellular compartments. (Left) The Cer-driven stress axis. Stress-induced *de novo* synthesis of Cer at the ER drives apoptosis. Pathological Cer accumulation initiates MOMP and the release of Cyt c, while concurrently activating PP2A to inhibit Bcl-2. (Right) The S1P-driven survival and epigenetic axis. The salvage pathway generates S1P via SphK1/2, establishing a bioenergetic network. Mitochondrial S1P interacts with PHB2 to enhance Complex IV respiration, while nuclear S1P inhibits HDAC1/2 to maintain an open chromatin state for the transcription of survival genes (*c-fos*, *p21*). Cytosolic S1P acts as an obligate cofactor for TRAF2 to trigger NF- κ B-mediated inflammatory responses. (Top) Intracellular S1P is exported via Spns2 (‘inside-out signalling’) to engage specific S1PR1–3, driving downstream survival and fibrotic cascades. This localised rheostat is integrated with E2 and FSH through the non-genomic activation of SphK1. Abbreviations: Cer, ceramide; S1P, sphingosine-1-phosphate; ER, endoplasmic reticulum; MOMP, mitochondrial outer membrane permeabilisation; Cyt c, cytochrome c; PP2A, protein phosphatase 2A; Bcl-2, B-cell lymphoma 2; SphK, sphingosine kinase; PHB2, prohibitin-2; HDAC, histone deacetylase; TRAF2, TNF receptor-associated factor 2; NF- κ B, nuclear factor kappa B; Spns2, Spinster-2; S1PR, sphingosine-1-phosphate receptor; E2, oestrogen; FSH, follicle-stimulating hormone. Figure 1. long description.

The essence of this rheostat lies in the dynamic interconvertibility and functional antagonism of these species. Ceramide, the central metabolic hub and its immediate derivative sphingosine are potent inhibitors of cell growth and established mediators of programmed cell death and fibrosis (Ref. 13). In stark contrast, their phosphorylated product, S1P, acts as a pleiotropic signalling molecule that promotes cell survival, migration, proliferation and angiogenesis. This metabolic pivot allows reproductive cells to rapidly reprogram their fate in response to microenvironmental cues, such as hormonal surges or oxidative stress. Consequently, a chronic shift in this equilibrium towards either ceramide accumulation or unchecked S1P signalling represents a molecular hallmark of various female reproductive disorders (Figure 1).

### Core metabolic pathways and enzymatic orchestration

The intracellular concentration of sphingolipid metabolites is governed by a highly compartmentalised and coordinated metabolic network that maintains a precise homeostatic balance between antagonistic bioactive species (Ref. 4).

#### Ceramide generation and metabolism

Ceramide, the central structural and signalling hub of the sphingolipid network, is tightly regulated via three distinct biosynthetic pathways (Figure 1):

- The *de novo* synthesis pathway: Occurring primarily in the endoplasmic reticulum, this pathway initiates with the rate-limiting condensation of L-serine and palmitoyl-CoA catalysed by serine palmitoyltransferase (SPT) (Ref. 7). Subsequent sequential reactions mediated by 3-ketodihydrosphingosine reductase and ceramide synthases (CerS1–6) – which exhibit distinct fatty acid chain-length specificities – yield dihydroceramide, which is ultimately desaturated by dihydroceramide desaturase (DES1/2) to form ceramide (Ref. 14).
- The sphingomyelin (SM) hydrolysis pathway: This rapid catabolic route involves the cleavage of membrane-bound SM by sphingomyelinases (SMases), notably the acid (aSMase/*Smpd1*) and neutral (nSMase/*Smpd2/3*) isoforms (Ref. 7). This pathway allows for the instantaneous, localised generation of ceramide across diverse subcellular compartments – including the plasma membrane, lysosomes and mitochondria – frequently in response to external environmental stressors or inflammatory cytokines.
- The salvage pathway: Complex glycosphingolipids undergo constitutive lysosomal degradation to regenerate ceramide, which is subsequently deacylated by ceramidases (CDases), particularly acid ceramidase (AC/*Asah1*), to yield sphingosine (Ref. 15). High basal AC activity is a critical prerequisite within the oocyte-granulosa microenvironment to prevent lipotoxic ceramide accumulation and subsequent germline apoptosis.

#### S1P generation and metabolism

The lifecycle of the pro-survival mediator S1P is orchestrated by a tightly balanced enzymatic rheostat governing its synthesis and catabolism:

- Biosynthesis: Free sphingosine generated from the salvage pathway is phosphorylated to form S1P by two distinct isoforms of sphingosine kinase: SphK1 and SphK2 (Ref. 16). SphK1 is primarily cytosolic and translocates to the plasma membrane upon growth-factor activation to drive ‘inside-out’ signalling, whereas SphK2 predominantly localises to the nucleus, mitochondria or ER, where it exerts context-dependent epigenetic or metabolic effects.
- Catabolism: Intracellular S1P levels are strictly capped to prevent unchecked proliferation via two distinct mechanisms: reversible dephosphorylation back to sphingosine by S1P phosphatases (SPP1/2 or SGPP1/2), or irreversible catabolic cleavage by endoplasmic reticulum-bound S1P lyase (SPL/*Sgpl1*) into hexadecenal and phosphoethanolamine (Ref. 11). Genetic loss or pathological downregulation of SPL results in aberrant intracellular S1P accumulation, disrupting the localised lipid gradients required for healthy germ cell maturation and follicular development.

#### S1P signalling dynamics: transporters and receptor heterogeneity

To execute its extracellular biological functions, intracellularly generated S1P must be exported across the plasma membrane, a process designated as ‘inside-out signalling’.

Vascular and Cellular Transporters: S1P export is strictly mediated by specialised transporters, predominantly Spinster-2 (Spns2) in endothelial/epithelial cells and MFSD2B in erythrocytes and platelets. These transporters establish the vital physiological S1P gradient between interstitial tissues (~0.1 μM in lymph) and systemic circulation (~1 μM in blood), which is indispensable for immune cell trafficking, localised cell migration and vascular barrier stability (Refs 10, 17).

G-Protein coupled Receptor (S1PR) Diversity: Extracellular S1P acts as a high-affinity ligand for five distinct G-protein coupled receptors (S1PR1–5). Within the female reproductive tract and its associated pathologies, S1PR1, S1PR2 and S1PR3 represent the dominant functional subtypes:

- S1PR1: Couples exclusively with G_i_ proteins to activate downstream PI3K/Akt/mTOR and Ras/MAPK/ERK pathways. This cascade drives cell survival, directed migration, and the structural stabilisation of VE-cadherin at endothelial and epithelial junctions (Ref. 18).
- S1PR2 and S1PR3: Exhibit multi-G-protein coupling capacity (G_i_, G_q_ and G_12/13_) (Ref. 19). Crucially, they serve as the primary upstream activators of the Rho/Rho-kinase (ROCK) signalling network. This specific pathway governs fibroblast-to-myofibroblast transdifferentiation (FMT), tissue contractility and growth cone collapse/sensitisation in pelvic pain states.
- S1PR4–5 and Endocrine Integration: While S1PR4 and S1PR5 are primarily restricted to hematopoietic and central nervous systems, their pathways cross-link heavily with the reproductive endocrine environment. Oestrogen (E2) and follicle-stimulating hormone rapidly stimulate SphK1 activation (Ref. 20). This lipid-hormone crosstalk triggers non-genomic oestrogenic effects, including acute intracellular calcium (Ca^2+^) surges and rapid ERK1/2 phosphorylation, which are mandatory for oocyte meiotic resumption and granulosa cell (GC) metabolic support.

### Intracellular second messenger signalling and organelle crosstalk

Beyond their canonical roles in membrane architecture and receptor-mediated signalling, sphingolipid metabolites function as critical intracellular second messengers that modulate diverse organelle-specific physiological and pathological programmes (Ref. 21).

#### Ceramide: a mediator of subcellular stress and apoptosis

Intracellular ceramide acts as a primary transducer of metabolic and environmental stress signals, localised predominantly within the mitochondria and endoplasmic reticulum (Figure 1).

- Mitochondrial outer membrane permeabilisation: Upon accumulation in the outer mitochondrial membrane, ceramide self-assembles into large-diameter, stable ceramide channels. These channels facilitate MOMP, driving the accelerated efflux of pro-apoptotic intermembrane space proteins (cytochrome c and SMAC/DIABLO) into the cytosol, thereby triggering the caspase-3 executioner cascade (Refs 22, 23).
- Bcl-2 Family Interplay and PP2A Activation: Intracellular ceramide promotes structural apoptosis by facilitating the conformational change and mitochondrial recruitment of Bax (Ref. 24). Concurrently, it activates mitochondrial protein phosphatase 2A (PP2A) (Ref. 25), which dephosphorylates and inactivates the anti-apoptotic rheostat Bcl-2, neutralising the follicle’s primary defense against atresia.
- ER Stress and Lipotoxicity: In metabolic reproductive conditions, ceramide bridges nutrient excess with organelle dysfunction. Elevated palmitic acid (PA) flux drives *de novo* ceramide accumulation, which induces severe ER stress and robustly upregulates CHOP expression, driving GC apoptosis and arresting oocyte developmental competence (Refs 26, 27).

#### S1P: a regulator of bioenergetics and epigenetics

In functional antagonism to ceramide, intracellular S1P operates as a vital survival factor by modulating mitochondrial metabolism, nuclear transcription and cytosolic inflammatory pathways (Figure 1).

- Mitochondrial Bioenergetics: SphK2-generated S1P within the inner mitochondrial membrane binds specifically to prohibitin-2 (PHB2) (Ref. 28). This interaction is required for the optimal assembly and catalytic activity of cytochrome c oxidase (Complex IV), enhancing mitochondrial respiration and ATP synthesis, which is crucial for fueling oocyte meiotic progression.
- Nuclear Epigenetic Programming: Within the nucleus, S1P binds to and inhibits histone deacetylases 1 and 2 (HDAC1/2) (Ref. 29). This inhibition blocks the removal of acetyl groups from histone tails, maintaining an open chromatin state that permits the transcription of pro-survival genes such as *p21* and *c-fos.* Additionally, nuclear S1P interacts with telomerase reverse transcriptase, enhancing its stability to support cellular longevity (Refs 29, 30).
- Cytosolic Pro-inflammatory Activation: SphK1-derived cytosolic S1P acts as an obligate cofactor for the E3 ubiquitin ligase TRAF2 (Ref. 31). This interaction promotes the polyubiquitination of RIP1, triggering NF-κB pathway activation (Ref. 32) and the subsequent transcription of pro-inflammatory cytokines and chemokines (e.g. IL-6, CCL5) that drive the microenvironmental remodelling characteristic of endometriosis and gynaecological malignancies.

## Sphingolipid and ovarian-centric disorders

Ovarian follicular fate – dormancy, growth or atresia – is tightly orchestrated by local signalling dynamics within the reproductive microenvironment. Disruption of the sphingolipid rheostat is closely associated with the pathogenesis of ovarian pathologies, typically characterised by an imbalance favouring pro-apoptotic ceramide accumulation over the protective S1P signalling axis (Ref. 7).

### Reproductive ageing and POI

**Figure 2.**
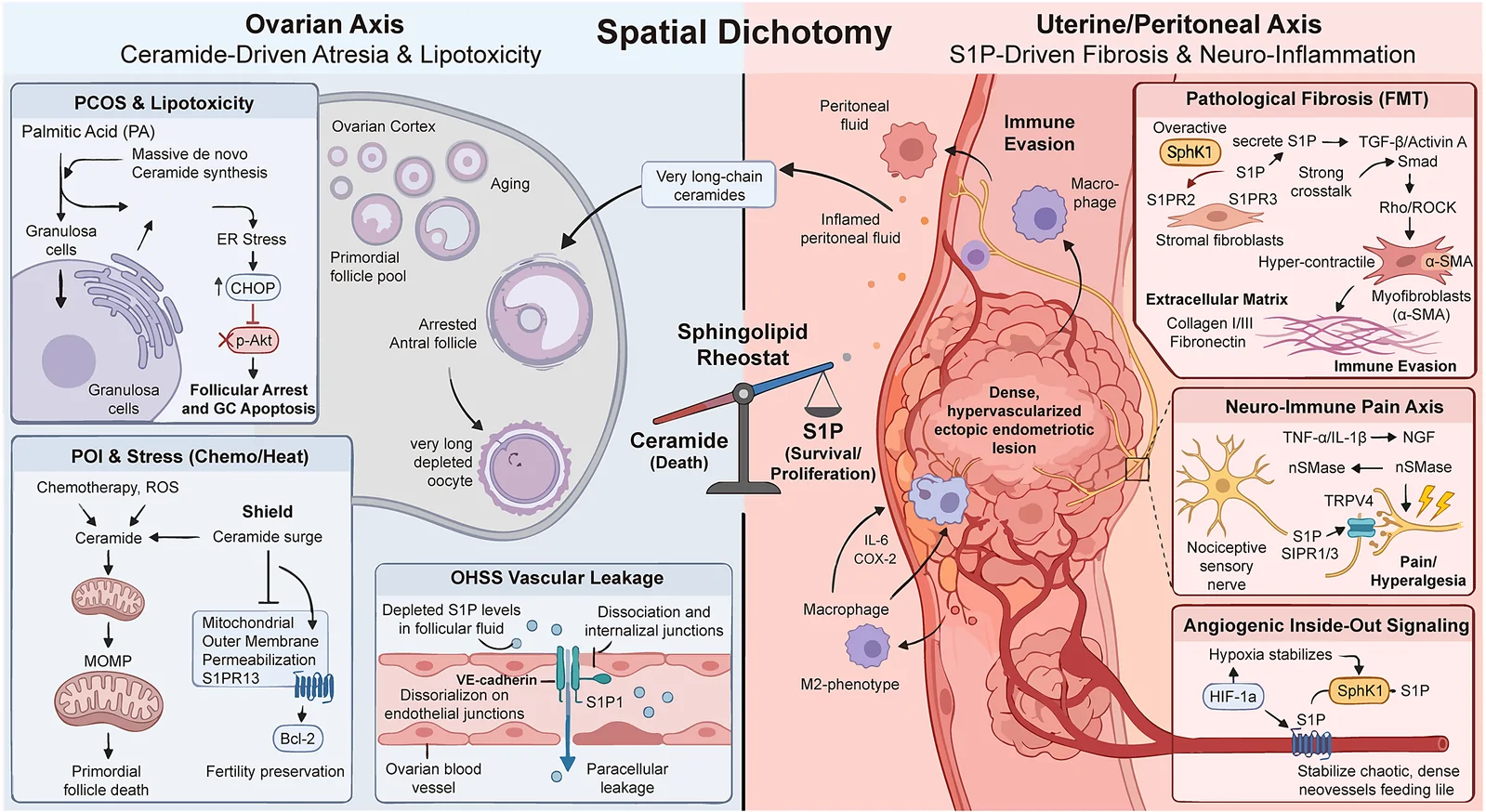
The spatial dichotomy of the sphingolipid rheostat in female reproductive pathologies. The sphingolipid metabolic axis exerts divergent, tissue-specific effects across the female reproductive continuum, acting as a molecular pivot between apoptotic atresia and hyper-proliferative survival. (Left Panel: Ovarian Compartment) A pathological shift toward Cer drives follicular depletion and vascular dysfunction. In PCOS, PA-induced Cer synthesis triggers ER stress via *CHOP*, blocking Akt signalling and causing follicular arrest. In POI and chemotoxicity, Cer surges initiate MOMP and germline apoptosis, a process that can be therapeutically shielded by exogenous S1P. Additionally, localised S1P depletion in OHSS destabilises VE-cadherin, leading to acute paracellular leakage. (Right Panel: Uterine and Peritoneal Compartment) Conversely, hyper-activation of the S1P axis drives pathological expansion in endometriosis and uterine fibroids. Extracellular S1P engages S1PR2/3 to activate the Rho/ROCK and TGF- β /Activin A pathways, inducing FMT and extensive ECM deposition. Concurrently, S1P promotes immune evasion by skewing macrophages towards an M2 phenotype and drives angiogenesis via the HIF-1α/S1PR1 axis. In the neuro-immune niche, NGF-stimulated S1P production sensitises nociceptive sensory neurons via TRPV4 channels, amplifying chronic pelvic pain. Bridging these two compartments, very long-chain ceramides from peritoneal endometriotic lesions exhibit retrograde toxicity, directly impairing adjacent oocyte competence. Abbreviations: Cer, ceramide; S1P, sphingosine-1-phosphate; PCOS, polycystic ovary syndrome; PA, palmitic acid; ER, endoplasmic reticulum; CHOP, CCAAT/enhancer-binding protein homologous protein; Akt, protein kinase B; POI, primary ovarian insufficiency; MOMP, mitochondrial outer membrane permeabilisation; OHSS, ovarian hyperstimulation syndrome; VE-cadherin, vascular endothelial cadherin; S1PR, sphingosine-1-phosphate receptor; ROCK, Rho-associated protein kinase; TGF- β , transforming growth factor-beta; FMT, fibroblast-to-myofibroblast transdifferentiation; ECM, extracellular matrix; M2, type 2 activated macrophage; HIF-1α, hypoxia-inducible factor 1-alpha; NGF, nerve growth factor; TRPV4, transient receptor potential vanilloid 4. Figure 2. long description.

Reproductive senescence entails a non-linear depletion of both the quantity and quality of the oocyte pool, culminating in the exhaustion of the ovarian reserve. Within this timeline, the sphingolipid network operates as both a distinctive molecular signature of cellular senescence and a direct mediator of follicular attrition (Ref. 8). This age-related acceleration of germ cell death is fundamentally driven by a rheostat shift towards pro-apoptotic pathways, which lowers the threshold of oocyte vulnerability to microenvironmental stressors (Figure 2).

#### Ceramide accumulation and subcellular mislocalisation

Oocyte apoptosis during ovarian ageing is an intercellular event reliant on biochemical crosstalk with surrounding somatic cumulus cells rather than an autonomous process. In senescent murine models, ceramide paradoxically accumulates within the cumulus cells while remaining low in basal-state oocytes (Ref. 33). Upon stress activation, this somatic ceramide is translocated into the adjacent oocyte via gap junctions and intact lipid rafts. This influx generates an aberrant accumulation of ceramide that disrupts homeostatic balance in the aged oocyte; unlike young oocytes that safely sequester exogenous ceramide within mitochondria to maintain cytoplasmic homeostasis, aged oocytes fail to do so, causing pathological lipid accumulation in the cytoplasm and plasma membrane (Ref. 34). Concurrently, the upregulation of *Bax* mRNA and Bax protein in senescent oocytes cooperates with this mislocalised ceramide to induce spontaneous cytoplasmic fragmentation and initiate the apoptotic cascade, demonstrating how altered intercellular lipid trafficking drives age-related fertility decline (Ref. 35).

#### Mitochondrial dysfunction and the MOMP pathway

Mitochondrial decay is a defining feature of age-related oocyte quality decline (Ref. 36), with ceramide serving as its principal executioner. Elevated outer mitochondrial membrane ceramide self-assembles into stable, macro-diameter ‘ceramide channels’, facilitating MOMP (Ref. 37). This permeabilisation triggers the accelerated efflux of cytochrome c and SMAC/DIABLO into the cytosol, activating the caspase-3 proteolytic cascade and irreversible cellular fragmentation (Refs 37, 38). Beyond disrupting membrane integrity, Cer actively suppresses metabolic survival pathways by activating protein phosphatase 2A (PP2A), which dephosphorylates and neutralises anti-apoptotic Bcl-2, thus lowering the oocyte’s apoptotic threshold (Ref. 39). Recent single-cell RNA-sequencing confirms that excess ceramide severely impairs developmental competence primarily via this organelle dysfunction (Ref. 40). Mechanistically, ceramide accumulation suppresses the intrafollicular AMPK/SIRT3 signalling axis, inducing mitochondrial protein hyperacetylation and metabolic collapse, a pathological state directly reproducible via exogenous ceramide administration in culture media (Ref. 41).

#### The role of ceramide transfer protein (CERT) and metabolism enzymes

Mitochondrial disintegration in senescent oocytes is structurally tied to the downregulation of CERT, which mediates non-vesicular ceramide transport from the endoplasmic reticulum to the Golgi or mitochondria (Ref. 42). Loss of CERT causes pathological lipid accumulation and organelle stress; notably, murine CERT mutations induce embryonic lethality via structural mitochondrial degeneration rather than canonical apoptosis, proving its necessity for early developmental organelle integrity (Ref. 43). This metabolic imbalance is compounded by the age-related loss of AC activity, which prevents the deacylation of ceramide into sphingosine for subsequent conversion into the pro-survival mediator S1P. Deficient AC activity shifts the metabolic profile towards elevated Bax/PARP expression and reduced Anti-Müllerian hormone levels, which is indicative of ovarian reserve depletion (Refs 44, 45). This enzymatic imbalance coincides with a cytotoxic microenvironment associated with the clinical manifestation of POI. Conversely, restoring AC expression or administering exogenous S1P neutralises ceramide toxicity, stabilises mitochondrial potential and rescues the developmental competence of aged oocytes, establishing these metabolic enzymes as viable therapeutic targets for expanding reproductive longevity.

### PCOS and metabolic lipotoxicity

PCOS – or polyendocrine-metabolic ovarian syndrome (PMOS) – is a prevalent systemic endocrine-metabolic disorder affecting up to 15% of reproductive-aged women globally (Refs 46, 47). Marked by disordered gonadotropin secretion, hyperandrogenism and chronic anovulation, PCOS is fundamentally underpinned by insulin resistance and obesity. A major component contributing to the accompanying reproductive failure is lipotoxicity, wherein ectopic lipid deposition within the ovarian compartment induces oxidative stress, worsens local insulin resistance and creates a pro-inflammatory follicular microenvironment that compromises oocyte developmental competence (Ref. 48) (Figure 2).

#### Dysregulation of sphingolipid metabolism

FF lipidomic profiling reveals profound sphingolipid remodelling in PCOS, though specific signatures vary across clinical phenotypes. Several cohorts exhibit a metabolic ‘silencing’ or downregulation of multiple FF sphingolipids – including ceramide, glucosylceramide and SM – which correlates with impaired oocyte development in assisted reproductive technology (ART) cycles (Refs 49, 50). Conversely, other subgroups display elevated systemic and local S1P, Cer and SM levels, pointing to potential regulatory links between circulating insulin or androgens, and altered bioactive lipid production (Ref. 51).

Advanced bioinformatic modelling (WGCNA and machine learning) of GC RNA-Seq datasets links this dysregulated sphingolipid metabolism directly to cellular senescence within the PCOS ovary (Ref. 52). These lipid aberrations, particularly within ceramide subclasses and lysophosphatidylglycerol (LPG), serve as sensitive markers of oocyte quality, with LPG concentrations significantly depleted in obese, insulin-resistant subgroups (Ref. 53). This disrupted landscape can be modulated therapeutically: metformin alters these aberrant sphingolipid profiles, while brown adipose tissue transplantation mitigates PCOS phenotypes by restoring lipid metabolites that regulate the PI3K/Akt and MAPK survival pathways (Refs 54, 55).

#### YAP signalling and follicular arrest

The hallmark follicular arrest of PCOS is tightly coupled to Hippo signalling pathway aberrations, where activation of the effector yes-associated protein (YAP) is driven by distorted follicular sphingolipid flux (Ref. 56). Physiologically, S1P disrupts Hippo signalling to stimulate GC proliferation and healthy follicular growth; thus, diminished S1P bioavailability or altered S1PR expression profiles promote the antral follicle arrest characteristic of PCOS. Pharmacologically, transcriptomic data suggest that low-dose aspirin may restore GC health and rescue follicular growth by normalising these senescence-associated sphingolipid pathways (Ref. 52).

#### Lipotoxicity, ER stress and granulosa cell viability

In obesity-complicated PCOS, ectopic accumulation of saturated free fatty acids, particularly PA, drives *de novo* ceramide synthesis within the ovary (Ref. 27). This ceramide surge triggers GC lipotoxicity, inducing severe endoplasmic reticulum stress marked by the robust upregulation of *CHOP* mRNA expression (Ref. 27). This lipotoxic environment compromises the insulin signalling cascade – evidenced by a suppressed phospho-Akt/Akt ratio – thereby decreasing cell viability and driving GC apoptosis. This ceramide-mediated ER stress-apoptosis axis provides a key pathological correlate linking systemic metabolic dysfunction to localised oocyte maturation failure in PCOS/PMOS patients.

### Ovarian and gynaecological cancers

#### Receptor dynamics in tumour expansion

The physiological versatility of the sphingolipid rheostat shifts towards oncogenesis in gynaecological malignancies, particularly ovarian cancer, where the balance between ceramide-mediated apoptosis and S1P-driven survival is altered (Figure 2). Malignant tissues reprogramme their metabolic machinery to upregulate SphK1 and SphK2, driving a marked depletion of pro-apoptotic ceramide and a concomitant surge in intracellular and extracellular S1P levels (Ref. 57). Clinically, S1P concentrations are significantly elevated in both the plasma and ascites of patients with epithelial ovarian cancer (Ref. 58), correlating strongly with advanced tumour stage, lymph node metastasis and poor overall survival (Ref. 59). Following surgical cytoreduction, these systemic S1P levels decline significantly, establishing this lipid mediator as both a critical oncogenic driver and a sensitive biomarker for disease recurrence (Ref. 58). Mechanistically, this oncogenic lipid shift accelerates tumour progression through multiple receptor-dependent pathways governing cell proliferation, migration and the epithelial-to-mesenchymal transition (EMT). Extracellular S1P, secreted into the tumour microenvironment, engages in ‘inside-out signalling’ primarily through S1PR1 and S1PR3, which are markedly overexpressed in malignant ovarian cells (Ref. 60). This receptor engagement couples with G_i_ and G_12/13_ proteins to potently stimulate downstream PI3K/Akt/mTOR and MAPK/ERK signalling cascades (Ref. 19), modulating cell cycle checkpoints and conferring a robust anti-apoptotic phenotype upon cancer cells.

Beyond tumour-intrinsic survival, the SphK/S1P axis is a regulator of tumour angiogenesis and neuro-angiogenic niches within the gynaecological tumour microenvironment (Ref. 61). S1P acts as a potent angiogenic factor that complements and frequently compensates for vascular endothelial growth factor (VEGF) signalling (Ref. 62). By binding to endothelial S1PR1, tumour-derived S1P promotes endothelial cell migration, capillary sprouting and neovessel stabilisation via pericyte recruitment (Ref. 63).

#### Chemoresistance paradigms

Mechanistically, this S1P signalling network underlies the molecular basis of chemoresistance to standard platinum- and taxane-based therapies (Ref. 64). While chemotherapeutic agents like cisplatin typically induce cellular stress to trigger a spike in *de novo* ceramide synthesis and initiate MOMP (Ref. 65), the overproduction of S1P effectively neutralises this ceramide accumulation, shifting the rheostat away from death and towards survival. This evasion of mitochondrial apoptosis presents a profound dilemma in oncofertility: while exogenous S1P administration holds immense therapeutic promise as an adjuvant to safeguard the primordial follicle pool against chemotherapy-induced gonadotoxicity, its systemic use carries the potential risk of protecting residual micrometastases or accelerating the recurrence of sphingolipid-dependent malignancies.

### Iatrogenic and environmental ovarian damage

The longevity and functional capacity of the ovarian reserve are constantly threatened by external perturbations, from gonadotoxic medical interventions to adverse environmental factors. The molecular node at the intersection of these disparate insults is the sphingolipid rheostat, which acts as a master sensor of follicle health versus atresia. Under homeostatic conditions, a high ratio of S1P to ceramide ensures cellular proliferation and survival within the follicular niche; however, exposure to iatrogenic or environmental stressors disrupts this equilibrium, tilting the molecular balance towards ceramide accumulation (Refs 66, 67). This metabolic shift initiates an intracellular cascade that couples lipid signalling with oxidative stress, ultimately accelerating germ cell elimination and driving premature ovarian insufficiency.

#### Chemotherapy-induced follicular atresia

The clinical management of malignancies in reproductive-aged women frequently involves cytotoxic agents, such as cyclophosphamide and doxorubicin, which deplete the primordial follicle pool by inducing massive apoptosis in both oocytes and surrounding GCs (Ref. 68). Mechanistically, these chemotherapeutic insults trigger a marked increase in cellular ceramide levels, mirroring core stress-induced cell death pathways across experimental models. This ceramide accumulation serves as an upstream signalling molecule that destabilises organelle integrity and commits the follicle to programmed atresia.

To counteract this gonadotoxic depletion, modulating the SphK-dependent seesaw represents a recognised approach in oncofertility. Exogenous S1P administration or intracellular SphK upregulation acts as a protective agent, safeguarding ovarian grafts and preserving the primordial pool. S1P mediates this cytoprotective effect by engaging its specific cell-surface G-protein coupled receptors, predominantly S1PR1 and S1PR3 (Ref. 69). Upon binding, S1P activates the downstream PI3K/Akt survival pathway, which upregulates the anti-apoptotic protein Bcl-2 and significantly reduces the recruitment of cleaved caspase-3-positive executioner cells within the ovarian cortex (Ref. 70). This receptor-mediated stabilisation effectively blocks chemotherapy-induced apoptosis, offering a viable strategy to preserve female fertility during oncological treatments.

#### Heat Stress (HS) and oxidative balance

Beyond iatrogenic interventions, environmental HS represents a profound physical disruptor of oocyte maturation, acting primarily through the subversion of the intrafollicular lipid profile. In porcine and bovine models, HS induces a marked upregulation of ceramide generation specifically within the GC layer of antral follicles, leaving the surrounding theca cells unaffected (Refs 8, 71). This localised collapse of the S1P-to-ceramide ratio serves as a critical pathological switch; the resulting ceramide accumulation triggers excessive production of reactive oxygen species, severe oxidative stress and extensive mitochondrial fragmentation within the maturing oocyte (Ref. 71).

The control of the ceramide/S1P balance by SphK is highly sensitive to the cellular redox state, where oxidative stress can paralyse the survival arm of the rheostat and lock the cell into a pro-apoptotic cycle (Ref. 67). Paradoxically, while physiological levels of S1P modulate normal redox signalling – such as engaging G_i_-coupled S1PR1 receptors to promote controlled superoxide and nitric oxide generation required for germ cells to achieve functional competency – a pathological shift due to environmental insults completely overwhelms this homeostatic window. When tilted towards ceramide, the rheostat functions as a primary determinant of oxidative stress-mediated cellular injury, analogous to ischemic and toxic tissue degradation pathways (Ref. 72). Pharmacological supplementation with S1P breaks this vicious cycle by restoring the structural and metabolic equilibrium within the follicle. By counteracting ceramide-mediated lipotoxicity, S1P stabilises the mitochondrial membrane potential, mitigates oxidative damage and restores the meiotic and developmental potential of immature oocytes subjected to thermal stress (Refs 8, 71).

### Ovarian hyperstimulation syndrome (OHSS)

#### Endothelial junction dissolution and localised S1P depletion

OHSS is a severe iatrogenic complication of controlled ovarian stimulation during ART, characterised by cystic ovarian enlargement and a significant systemic increase in capillary permeability (Ref. 73). The central pathophysiological hallmark of OHSS is an acute fluid shift from the intravascular compartment into third spaces (peritoneal and pleural cavities), driving ascites, pleural effusion and hemoconcentration. At the molecular level, this vascular hyperpermeability is tied to the disruption of endothelial barrier integrity, where the sphingolipid network acts as a key regulator of endothelial junctions (Ref. 74). Physiologically, vascular S1P regulates endothelial stability by engaging cell-surface S1PR1 receptors to promote the assembly and localised retention of VE-cadherin at cell–cell appositions, thereby reinforcing the cortical actin cytoskeleton and sealing the paracellular barrier against unwanted fluid exudation.

In patients with severe OHSS, this protective endothelial shield coincides with a significant localised reduction of S1P concentrations within the FF microenvironment. This S1P deficiency deprives endothelial cells of an essential stabilising signal, leaving the ovarian and peritoneal vasculature highly vulnerable to hyperpermeability factors that run unchecked during hyperstimulation, most notably VEGF. The consequent loss of S1P-mediated S1PR1 signalling leads to the destabilisation and internalisation of VE-cadherin complexes, facilitating endothelial barrier dissolution, cellular fenestration and paracellular leakage (Ref. 75) (Figure 2).

#### Therapeutic restoration of capillary barrier integrity

Demonstrating the therapeutic potential of targeting this lipid node, preclinical interventions show that restoring S1P bioavailability effectively rescues the vascular phenotype. Specifically, *in vivo* intrabursal administration of S1P into the ovarian microenvironment significantly increases vascular integrity in rat models of OHSS (Ref. 76). This localised delivery of exogenous S1P counteracts systemic and local capillary leakage, significantly reducing peritoneal fluid (PF) accumulation, normalising ovarian weight and reversing hyperstimulation-related vascular disorders (Ref. 76). Collectively, these insights underscore that local S1P depletion is a critical permissive step in OHSS-associated vascular breakdown; its pharmacological reactivation offers a powerful, non-hormonal strategy to preserve vascular integrity and mitigate the clinical severity of ovarian hyperstimulation.

## Sphingolipid and uterine and endometrial disorders

### Endometriosis

While ovarian dysregulation of the sphingolipid rheostat typically promotes atresia, its manifestation within uterine and peritoneal microenvironments drives survival, pathological fibrosis and immune remodelling. Endometriosis is an oestrogen-dependent inflammatory disease characterised by ectopic endometrial-like stroma and glands (Ref. 77). Metabolomic analyses reveal systemic dysregulation in patients, marked by elevated circulating fatty acyl groups and ceramides (Ref. 78). Paradoxically, within both eutopic and ectopic endometrium, upregulated SMases decrease SM levels to inhibit apoptosis (Ref. 79). This survival phenotype is supported by accumulated local SM and phosphatidylcholine subspecies associated with altered lipid signalling during ectopic implantation (Ref. 80).

The hyper-activated S1P signalling axis serves as a prominent hallmark of these invasive features, mediating neuropeptide S-induced cell migration and lesion expansion (Ref. 81). Notably, ectopic lesions exert a retro-axial pathogenic effect on the ovary, driving altered autophagy in GCs and elevating very long-chain ceramides within the PF (Ref. 10). These peritoneal ceramides act as lipotoxic agents that disrupt neighbouring oocyte meiotic maturation, highlighting a strong correlation between pelvic lipid dysregulation and subfertility. This retrograde metabolic crosstalk underlines how localised pelvic lesions project lipotoxic damage back onto the ovarian niche, compromising the oocyte microenvironment (Figure 2).

#### Peritoneal evasion and immune remodelling

Concurrently, PF accumulation of S1P drives local immune evasion and microenvironmental remodelling. This peritoneal S1P surge functions as a chemokine that recruits monocytes and macrophages while preventing their apoptosis via constitutive PI3K/Akt and ERK1/2 activation (Ref. 82). Crucially, S1P skews these cells towards an immunosuppressive M2 macrophage phenotype (Ref. 83). These M2 macrophages establish an inflammatory loop: S1P stimulates their expression of IL-6 and cyclooxygenase-2 (COX-2), promoting chronic pelvic inflammation and lesion growth (Ref. 84).

#### Pathological fibrosis and fibroblast-to-myofibroblast transdifferentiation

Pathological fibrosis is an irreversible feature of endometriotic progression that causes severe pelvic scarring and chronic pain. The SphK1/S1P/S1PRs axis is closely involved in this fibrotic engine by synergising with canonical TGF-β1/Smad signalling (Ref. 85). Locally elevated S1P potently stimulates FMT, prompting stromal fibroblasts to express α-smooth muscle actin (α-SMA) and secrete excessive extracellular matrix (ECM) proteins like collagen and fibronectin (Ref. 86). This FMT process is primarily orchestrated by S1PR2 and S1PR3 coupling with G_12/13_ proteins to activate downstream Rho/ROCK signalling (Ref. 87). Rho/ROCK activation increases myofibroblast contractility and tissue stiffness, maintaining the lesion in a fibrotic state.

#### Angiogenic ‘Inside-Out’ signalling

To sustain its hyper-proliferative phenotype, the ectopic lesion establishes a persistent blood supply via angiogenic ‘inside-out signalling’. Unlike physiological angiogenesis, endometriotic vascularisation is chaotic, excessive and resistant to pruning. While VEGF initiates premature microvessel sprouting, S1PR1 is indispensable for structural stabilisation (Ref. 88). Lesion-exported S1P binds endothelial S1PR1, consolidating VE-cadherin at cell–cell junctions to form a functional vascular barrier. Within the expanding lesion, local hypoxia stabilises HIF-1α, which directly upregulates SphK1 (Ref. 89). This hypoxia-induced enzymatic activation creates a feed-forward loop that ensures a continuous supply of S1P to the peritoneal microenvironment, stabilising the vascular network and supporting sustained ectopic growth.

### Adenomyosis and uterine fibroids

Both adenomyosis and uterine fibroids are benign, oestrogen-responsive gynaecological disorders characterised by severe dysregulation of the S1P axis, which promotes aberrant smooth muscle cell proliferation, ECM deposition and pathological tissue rigidity (Figure 2).

#### Uterine adenomyosis

Uterine adenomyosis, characterised by the invasion of endometrial glands and stroma into the myometrium, exhibits a heavily distorted sphingolipid profile (Ref. 90). Ectopic lesions selectively upregulate the S1P3 receptor subtype compared to healthy endometrium (Ref. 90). This dysregulation links directly to TGF-β1 signalling, where S1P3 acts as a non-hormonal transducer that amplifies TGF-β-induced fibrotic cascades (Ref. 91). Hyper-activation of the S1P/S1P3 axis correlates with the robust transcriptional activation of *ACTA2* (encoding α-smooth muscle actin), driving the fibrotic transdifferentiation of endometrial stromal cells.

This fibrotic remodelling exhibits distinct stage-dependent and postmenopausal dynamics. While adenomyosis typically regresses symptomatically after menopause due to oestrogen withdrawal, α-SMA expression and tissue fibrosis can paradoxically increase in persistent postmenopausal lesions (Ref. 92). This progression is closely associated with defective S1P catabolism – marked by the downregulation of S1P lyase or S1P phosphatases – and altered downstream signalling, which locks the senescent myometrial microenvironment into a chronic fibrotic state (Ref. 92).

In tandem with structural stiffening, defects in the opposing arm of the sphingolipid rheostat enable adenomyotic cells to survive within the myometrium. Eutopic and ectopic stromal cells exhibit a severely damaged ceramide apoptotic pathway. When exposed to cellular stress or pro-apoptotic cues, these pathologically altered cells fail to accumulate ceramide or initiate mitochondrial channel formation, demonstrating a robust resistance to canonical apoptosis that facilitates deep myometrial infiltration (Ref. 93). Given these temporal fluctuations across disease progression, implementing a stage-dependent therapeutic strategy using S1P3-selective antagonists offers a promising, non-hormonal approach for long-term adenomyosis management.

#### Uterine fibroids

Uterine fibroids (leiomyomas) are benign clonal tumours characterised by massive myometrial smooth muscle cell expansion and disproportionate ECM accumulation. Molecular profiling confirms extensive dysregulation of the SphK/S1P/S1PR axis in leiomyoma tissues compared to adjacent healthy myometrium. Quantitatively, mRNA and protein levels of SphK1, SphK2 and receptors S1P_2_, S1P_3_ and S1P_5_ are significantly elevated within fibroid nodules (Refs 94, 95). This localised enzymatic hyper-activation shifts the lipid microenvironment, allowing S1P to act as a potent, constitutive proliferative and fibrotic cue that drives leiomyoma growth and tissue stiffening.

A central mechanism of this fibrotic phenotype is the synergistic crosstalk between S1P and Activin A, a member of the TGF-β superfamily. Activin A expression is markedly upregulated in uterine fibroids, establishing a pro-fibrotic microenvironmental niche (Ref. 95). Secreted S1P serves as an essential downstream mediator and amplifier of Activin A-induced cascades, engaging overexpressed S1PRs to activate downstream Smad and non-Smad pathways. This receptor-mediated activation functionalises a feed-forward loop with Activin A, significantly enhancing the transcription and deposition of key ECM structural proteins, including collagen type I, collagen type III and fibronectin (Refs 95, 96).

Furthermore, the S1P-Activin A axis contributes directly to the structural rigidity of leiomyomas by promoting cellular hypertrophy and mechanosensitive adaptation. Hyper-activation of S1P_2_ and S1P_3_ receptors triggers downstream G-protein-coupled Rho/ROCK signalling, leading to stress fibre formation, increased cellular tension and the subsequent activation of mechanosensitive gene transcription (Ref. 94). This lipid-dependent structural stabilisation provides a basis for understanding how fibroids maintain matrix remodelling independent of canonical steroid hormone pathways. Targeting the SphK/S1P/S1PRs-Activin A axis represents a highly promising, non-hormonal therapeutic strategy that treats fibroids without compromising the systemic endocrine baseline (Refs 95, 96).

### Endometrial receptivity and implantation

Bioactive sphingolipids and their phospholipid derivatives serve as indispensable homeostatic regulators required for the successful establishment of early pregnancy, orchestrating synchronised maternal–embryonic crosstalk during the peri-implantation window (Refs 97, 98). Dynamic lipidomic profiling reveals that localised lipid metabolism undergoes precise spatio-temporal reprogramming to dictate uterine receptivity, blastocyst attachment and subsequent decidualisation (Ref. 98). A foundational element of this uterine preparation is the tightly regulated fluctuation of sphingolipids within the endometrial stroma. In murine models, embryo implantation triggers a synchronous surge in local ceramide levels, strictly coupled with the transcriptional upregulation of the acid SMase gene (*Smpd1*) at active implantation sites (Ref. 99). This coordinated enzymatic activation ensures rapid, localised ceramide generation from SM hydrolysis, providing the membrane fluidity and signalling platforms necessary for initial embryo apposition. Conversely, genetic or pathological impairments in new SM synthesis disrupt this specialised lipid landscape, causing profound decidualisation defects, compromised placental bed formation and early pregnancy loss (Ref. 100).

Central to this early gestational cascade is S1P, which functions as a master coordinator of early pregnancy dynamics. Translating evidence from animal models and human clinical cohorts associates S1P signalling with the molecular execution of endometrial receptivity, optimal interembryonic spacing along the uterine horns and the initiation of uterine stromal decidualisation (Ref. 9). Beyond organising the maternal compartment, S1P plays a vital paracrine role in guiding trophoblast behaviour and driving placental vascularisation. Secreted S1P acts through specific receptor networks to regulate the directional migration and proliferation of the invading chorionic villus trophoblast layer, anchoring the developing placenta to the maternal uterine wall while simultaneously promoting localised angiogenesis (Ref. 97). Given this absolute dependency on precise S1P gradients, either a localised deficiency in S1P synthesis or its pathological overproduction can disrupt uterine receptivity required for embryonic acceptance, presenting a cryptic, non-hormonal factor associated with recurrent implantation failure.

The pathological consequences of disrupting this sphingolipid-dependent pathway are clearly illustrated in chronic endometritis. Chronic inflammatory stress triggers severe endoplasmic reticulum stress within the endometrial epithelium, driving the aberrant activation of the transcription factor CCAAT/enhancer-binding protein delta (CEBPD) (Ref. 101). Once activated, CEBPD protein transcriptionally upregulates the S1P phosphatase 2 (*SGPP2*) gene, leading to S1P degradation. This pathological overexpression of SGPP2 causes an acute local exhaustion of S1P signalling, dismantling the essential receptor-mediated pathways required for physiological vascular remodelling and endothelial barrier stabilisation during the secretory phase. This inflammation–ER stress–*SGPP2* axis induces a failure of endometrial receptivity and structural decidualisation, providing a direct mechanistic link bridging localised chronic inflammation to endometritis-related reproductive failure (Ref. 101).

### Chronic pain and neuro-immune crosstalk

Chronic pelvic pain is the most debilitating symptom in patients with gynaecological disorders like endometriosis and provoked vestibulodynia, severely compromising quality of life and sexual function. Emerging translational evidence establishes the sphingolipid axis as a key mediator of pain amplification bridging localised inflammation and pathological neuro-immune crosstalk (Ref. 102). Within the pelvic microenvironment, ectopic lesion-derived inflammatory cytokines, such as TNF-α and IL-1β, stimulate the robust release of nerve growth factor (NGF) (Ref. 103). Mechanistically, localised ceramide accumulation induces thermal hyperalgesia by stimulating a reciprocal increase in tissue TNF-α and obligating the recruitment of the downstream S1P-to-S1PR1 signalling pathway (Figure 2).

Once generated within this neuro-immune niche, S1P engages S1PR1 and S1PR3 receptors on primary afferent sensory neurons to profoundly lower their mechanical and thermal excitation thresholds, driving clinical allodynia and hyperalgesia. This axis amplifies persistent pain by cross-linking with nociceptive transient receptor potential (TRP) channels, notably functionalising the S1P/S1PRs–TRPV4 axis to gate pro-nociceptive ion fluxes and perpetuate chronic mechanical hypersensitivity (Ref. 104). Furthermore, this lipid-driven nociceptive signature is systemic and phenotype-dependent. Circulating ceramides and SMs are significantly elevated in obese cohorts, correlating with pain severity and linking metabolic obesity to chronic musculoskeletal and pelvic pain syndromes (Ref. 105). Disruption of these sphingolipid pathways directly associates with vulvar pain, levator ani muscle tenderness and altered functional connectivity within pain-processing brain regions, while early-life pain experiences can cause a long-term reduction in long-chain sphingolipids that correlates with later compulsive pain behaviours (Ref. 102).

In tandem with functional peripheral sensitisation, the sphingolipid rheostat governs the structural remodelling of the pelvic nervous system via pathological innervation dynamics. Within deep infiltrating endometriotic lesions, S1P exerts a complex, receptor-dependent dual effect on neurogenesis and nerve sprouting. Activation of neuronal S1PR1 potently promotes axonal elongation and the directional sprouting of unmyelinated sensory nerve fibres towards inflammatory foci. Conversely, when S1P engages S1PR3 on the same growth cones, it couples with G_12/13_ proteins to trigger downstream RhoA/ROCK pathway activation, mediating growth cone collapse and localised neurite retraction (Ref. 87). The localised imbalance between S1PR1-mediated elongation and S1PR3-mediated growth cone steering leads to highly disorganised, hyper-dense and hypersensitive sensory nerve networks within the subperitoneal space. This aberrant neurovascular niche remains locked in a state of persistent activation, rendering the S1P axis a promising, non-hormonal therapeutic target to disrupt the feed-forward cycle of neuro-immune inflammation and chronic pelvic pain.

## Clinical translation and future perspectives

The multi-layered involvement of the sphingolipid rheostat in both physiological reproduction and gynaecological pathologies presents significant clinical opportunities. Shifting the paradigm from systemic hormonal suppression – which routinely induces hypoestrogenic side effects and halts ovulation – to targeted lipid interventions offers a non-hormonal frontier for precision diagnosis and therapeutic management.

### Sphingolipid metabolites as fluid-based diagnostic biomarkers

The state-dependent fluctuations of ceramide and S1P within plasma and FF mirror the real-time pathophysiological status of the reproductive microenvironment, serving as sensitive candidates for non-invasive biomarker discovery. In ART, lipidomic fingerprinting provides critical predictive insights into ovarian reserve and individual cycle outcomes. In women with diminished ovarian reserve or reproductive ageing, quantitative assessments of serum and FF ceramide levels serve as independent indicators of oocyte quality and *in vitro* fertilisation success (Ref. 106). Furthermore, FF profiling from patients with unexplained infertility reveals a distinct phenotype marked by enriched triacylglycerols and severe depletion of functional phospholipids and sphingolipids (Ref. 107). This discriminative signature links local sphingolipid deficits to a suboptimal follicular niche, offering an innovative diagnostic layer to identify metabolic etiologies of reproductive failure prior to controlled ovarian stimulation.

Beyond predicting ART outcomes, specific sphingolipid subclasses furnish disease-specific signatures that enable early detection and risk stratification. In PCOS, a coordinated downregulation of multiple FF sphingolipid species and phosphatidylcholines correlates with gonadotropin hyper-responsiveness and impaired oocyte maturation (Ref. 50). Conversely, advanced epithelial ovarian cancer features a profound accumulation of circulating and tissue-level long-chain ceramides, SMs and glycolipids, which drop precipitously following surgical cytoreduction, serving as sensitive diagnostic and staging biomarkers (Ref. 108).

In benign proliferative disorders, targeted metabolomics demonstrates that pelvic endometriosis is characterised by elevated systemic and local SM and phosphatidylcholine levels (Ref. 80). Crucially, high-resolution liquid chromatography discriminates between generalised peritoneal endometriosis and localised ovarian endometriomas based on unique mass-to-charge ratio (m/z) signatures of their respective lipid pools, providing an advanced tool for differential diagnosis. Finally, in acute vascular emergencies like OHSS, a precipitous collapse of pre-ovulatory FF S1P levels acts as an early biomonitoring index predicting endothelial junction breakdown and severe vascular hyperpermeability (Ref. 76). Together, these multi-disease lipid signatures pave the way for incorporating targeted sphingolipid panels into standard gynaecological triage and personalised reproductive medicine.

### Therapeutic frontiers: modulating the sphingolipid network for fertility preservation

The transition of the sphingolipid rheostat from a diagnostic tool to a therapeutic target represents a major frontier in non-hormonal gynaecological pharmacology. Because the balance between ceramide-mediated apoptosis and S1P- or ceramide-1-phosphate (C1P)-mediated survival dictates cell fate, interventions designed to selectively tilt this rheostat offer unprecedented strategies for restoring ovarian health. This approach is vital in oncofertility, where safeguarding the primordial follicle pool against gonadotoxic insults remains a primary clinical challenge.

In this context, C1P – synthesised via ceramide kinase – functions as a potent cytoprotective agent within the ovarian niche. Preclinical evidence in murine models of POI demonstrates that exogenous C1P administration exerts robust protective properties against cyclophosphamide-induced ovarian damage (Ref. 109). Mechanistically, C1P neutralises the excessive *de novo* ceramide accumulation triggered by alkylating chemotherapeutic agents, thereby suppressing GC apoptosis, preserving ovarian cortical integrity, and maintaining baseline follicle pool viability. Targeting specific bioactive derivatives like C1P provides a promising, receptor-specific framework to counteract iatrogenic ovarian insufficiency and extend female reproductive longevity.

### Current limitations and future directions

Despite the substantial diagnostic and therapeutic potential of the sphingolipid metabolic network, several critical bottlenecks and clinical hurdles must be resolved before these lipid-targeted interventions can transition into standard gynaecological practice.

The primary pharmacological challenge stems from the inherent conflict between receptor ubiquity and systemic toxicity. Because sphingosine-1-phosphate receptors (S1PR1–5) are ubiquitously expressed across the cardiovascular and immune systems, broad-spectrum lipid modulators carry a high risk of inducing severe off-target effects, such as bradycardia and profound immunosuppression. To bypass these systemic liabilities, future drug discovery pipelines must prioritise the design of next-generation, highly selective sub-receptor antagonists – such as JTE013 for S1P2 or TY-52156 for S1P3. These subtype-specific small molecules hold the key to precisely dismantling localised pelvic fibrotic and nociceptive pathways without disrupting systemic homeostatic functions.

A secondary clinical dilemma involves establishing the long-term oncological safety of these survival-promoting lipids within oncofertility. Because S1P is inherently anti-apoptotic and angiogenic, its application as a pharmacological shield to safeguard the primordial follicle pool against gonadotoxic chemotherapy introduces a severe risk of inadvertently protecting residual micrometastases or accelerating tumour recurrence.

Furthermore, a major methodological challenge in reproductive lipidomics lies in delineating whether the altered sphingolipid profiles observed in clinical cohorts represent primary upstream drivers of pathology or secondary downstream manifestations of systemic metabolic dysfunction. The majority of current human dataset matrices rely on cross-sectional clinical associations, which capture correlative biomarker signatures rather than direct causal networks. While these fluid-based profiles provide valuable predictive indicators for clinical triage, establishing absolute pathogenic causality requires validation through functional loss- or gain-of-function transgenic animal models. Disentangling these associative metabolic reflections from authentic molecular etiologies remains a prerequisite for the accurate identification of therapeutic targets.

Finally, navigating the context-dependent, pleiotropic nature of sphingolipid flux demands a transition towards high-resolution mechanistic mapping within the reproductive tract. S1P signalling is highly dynamic, capable of driving either pro-inflammatory M1 or immunosuppressive M2 macrophage polarisation depending on localised cytokine cues. Consequently, relying on bulk tissue lysates no longer suffices. The future of this field depends on integrating single-cell multi-omics – including transcriptomics and proteomics – with high-resolution spatial metabolomics and mass spectrometry imaging. Unlocking this spatial and cellular granularity is an absolute prerequisite to accurately map localised lipid fluxes across distinct cellular subpopulations, transforming our understanding of the sphingolipid network into a predictable, finely controlled therapeutic landscape for female reproductive health.

## Conclusion

The sphingolipid rheostat acts as a fundamental molecular switch in the female reproductive system, balancing the balance between cell survival and apoptosis. Disruptions in this lipid network lie at the pathogenic core of a wide spectrum of disorders, from ovarian ageing and PCOS to the fibrotic and inflammatory landscapes of endometriosis and uterine fibroids. By decoding these intricate metabolic pathways, the field is poised to unlock novel, targeted therapeutic strategies that not only alleviate debilitating symptoms but also preserve and restore reproductive longevity.

## Long descriptions

### Figure 1. Long description

The diagram is divided into two main vertical zones.

Left Zone: Ceramide and Apoptosis Death Axis.

* At the top, the Endoplasmic Reticulum (E R) shows L-serine and Palmitoyl-C o A being converted by S P T and Cer S 1-6 into Ceramide.

* Below, Ceramide interacts with a mitochondrion, forming Ceramide Channels that lead to Catastrophic Cytochrome c release and Caspase-3 activation.

* To the far left, P P 2 A inhibits Bcl-2 to promote apoptosis.

Right Zone: S 1 P and Survival Axis.

* At the center, Ceramide is converted to S 1 P via Sph k 1 and Sph k 2.

* In the mitochondrion, S 1 P interacts with P H B 2 and Complex I V to produce A T P.

* In the nucleus, S 1 P inhibits H D A C 1/2, maintaining open chromatin for pro-survival genes like c-fos and p21.

* In the cytosol, S 1 P binds T R A F 2 to activate N F-kappa B.

Top Perimeter: Plasma Membrane and Signaling.

* S 1 P is exported via Spns 2 (Inside-Out Signaling) to bind extracellular receptors S 1 P R 1 and S 1 P R 2/3.

* S 1 P R 1 activates P I 3 K/Akt and M A P K/E R K pathways.

* S 1 P R 2/3 activates Rho/R O C K for cytoskeletal changes.

* On the far right, E 2 and F S H receptors trigger rapid non-genomic activation of Sph k 1.

Navigate back to Figure 1..

### Figure 2. Long description

At the center is a balance scale labeled Sphingolipid Rheostat, with Ceramide (Death) on the left weighing down and S 1 P (Survival/Proliferation) on the right.

Left Panel (Ovarian Axis):

- Top left: P C O S and Lipotoxicity. Palmitic Acid (P A) leads to massive de novo Ceramide synthesis in granulosa cells, triggering E R Stress, C H O P activation, and blocking p-Akt, resulting in follicular arrest and G C apoptosis.

- Middle left: Ovarian Cortex diagram showing the transition from a primordial follicle pool to an arrested antral follicle and a very long depleted oocyte due to aging.

- Bottom left: P O I and Stress. Chemotherapy and R O S cause a Ceramide surge leading to M O M P and primordial follicle death. A ‘Shield’ pathway shows S 1 P R 1 3 and Bcl-2 inhibiting this process for fertility preservation.

- Bottom center: O H S S Vascular Leakage. Depleted S 1 P levels in follicular fluid lead to V E-cadherin dissociation on endothelial junctions and paracellular leakage in ovarian blood vessels.

Right Panel (Uterine/Peritoneal Axis):

- Center: A large, dense, hypervascularized ectopic endometriotic lesion.

- Top right: Pathological Fibrosis (F M T). Overactive SphK 1 and S 1 P R 2/3 signaling in stromal fibroblasts activate T G F-beta/Activin A and Rho/R O C K pathways, leading to alpha-S M A myofibroblast formation and E C M deposition (Collagen I/III, Fibronectin).

- Middle right: Neuro-Immune Pain Axis. T N F-alpha and I L-1 beta stimulate N G F, which activates nSMase and S 1 P. S 1 P engages S 1 P R 1/3 and T R P V 4 on nociceptive sensory nerves, causing pain and hyperalgesia.

- Bottom right: Angiogenic Inside-Out Signaling. Hypoxia stabilizes H I F-1 a, which upregulates SphK 1 to produce S 1 P, stabilizing chaotic neovessels feeding the lesion.

- Peritoneal Fluid: Shows immune evasion where macrophages are skewed to an M 2-phenotype by I L-6 and C O X-2.

- A bridging arrow shows very long-chain ceramides from the peritoneal fluid moving toward the ovarian cortex to impair oocytes.

Navigate back to Figure 2..

## References

[1] Yao S, Lopez-Tello J and Sferruzzi-Perri AN (2021) Developmental programming of the female reproductive system – A review. Biology of Reproduction 104(4), 745–770. 10.1093/biolre/ioaa232. 33354727

[2] Zhang X, Cao Q, Rajachandran S, Grow EJ, Evans M and Chen H (2023) Dissecting mammalian reproduction with spatial transcriptomics. Human Reproduction Update 29, 794–810. 10.1093/humupd/dmad017. PMC10628492 37353907

[3] Winkler I, Tolkachov A, Lammers F, Lacour P, Daugelaite K, Schneider N, Koch ML, Panten J, Grünschläger F, Poth T, Ávila BM, Schneider A, Haas S, Odom DT and Gonçalves Â (2024) The cycling and aging mouse female reproductive tract at single-cell resolution. Cell 187, 981.e25–998.e25. 10.1016/j.cell.2024.01.021. 38325365

[4] Hannun YA and Obeid LM (2018) Sphingolipids and their metabolism in physiology and disease. Nature Reviews. Molecular Cell Biology 19(3), 175–191. 10.1038/nrm.2017.107. PMC5902181 29165427

[5] Gruevska A, Leslie J, Perpiñán E, Maude H, Collins AL, Johnson S, Evangelista L, Sabey E, French J, White S, Moir J, Robinson SM, Alrawashdeh W, Thakkar R, Forlano R, Manousou P, Goldin R, Carling D, Hoare M, Thursz M, Mann DA, Cebola I, Posma JM, Safinia N, Oakley F and Hall Z (2025) Spatial lipidomics reveals sphingolipid metabolism as anti-fibrotic target in the liver. Metabolism: Clinical and Experimental 168, 156237. 10.1016/j.metabol.2025.156237. 40127860

[6] Fernandis AZ and Wenk MR (2007) Membrane lipids as signaling molecules. Current Opinion in Lipidology 18(2), 121–128. 10.1097/MOL.0b013e328082e4d5. 17353659

[7] Tabasi R, Ghasemian F and Tavana S (2025) Sphingolipids as key mediators in folliculogenesis and female fertility. Life Sciences 374, 123697. 10.1016/j.lfs.2025.123697. 40348177

[8] Hernández-Coronado CG, Guzmán A, Castillo-Juárez H, Zamora-Gutiérrez D and Rosales-Torres AM (2019) Sphingosine-1-phosphate (S1P) in ovarian physiology and disease. Annales D’endocrinologie 80(5-6), 263–272. 10.1016/j.ando.2019.06.003. 31455516

[9] Yang T, Zhao J, Liu F and Li Y (2022) Lipid metabolism and endometrial receptivity. Human Reproduction Update 28(6), 858–889. 10.1093/humupd/dmac026. 35639910

[10] Turathum B, Gao E-M, Grataitong K, Liu YB, Wang L, Dai X and Chian RC (2022) Dysregulated sphingolipid metabolism and autophagy in granulosa cells of women with endometriosis. Frontiers in Endocrinology 13, 906570. 10.3389/fendo.2022.906570. PMC9381821 35992117

[11] Gault CR, Obeid LM and Hannun YA (2010) An overview of sphingolipid metabolism: From synthesis to breakdown. In Chalfant C and Del Poeta M (eds.), Sphingolipids as Signaling and Regulatory Molecules, *Advances in Experimental Medicine and Biology* 688. New York: Landes Bioscience, pp. 1–23. 10.1007/978-1-4419-6741-1_1. PMC3069696 20919643

[12] Bartke N and Hannun YA (2009) Bioactive sphingolipids: Metabolism and function. Journal of Lipid Research 50(Suppl.), S91–S96. 10.1194/jlr.R800080-JLR200. PMC2674734 19017611

[13] Castro BM, Prieto M and Silva LC (2014) Ceramide: A simple sphingolipid with unique biophysical properties. Progress in Lipid Research 54, 53–67. 10.1016/j.plipres.2014.01.004. 24513486

[14] Mostafa S, Nader N and Machaca K (2022) Lipid signaling during gamete maturation. Frontiers in Cell and Developmental Biology 10, 814876. 10.3389/fcell.2022.814876. PMC9531329 36204680

[15] Birbes H, El Bawab S, Hannun YA and Obeid LM (2001) Selective hydrolysis of a mitochondrial pool of sphingomyelin induces apoptosis. FASEB Journal: Official Publication of the Federation of American Societies for Experimental Biology 15(14), 2669–2679. 10.1096/fj.01-0539com. 11726543

[16] Gomez-Larrauri A , Presa N , Dominguez-Herrera A , Ouro A , Trueba M and Gomez-Muñoz A (2020) Role of bioactive sphingolipids in physiology and pathology. Essays in Biochemistry 64(3), 579–589. 10.1042/EBC20190091. 32579188

[17] Zhang F and Lu Y (2023) The sphingosine 1-phosphate axis: An emerging therapeutic opportunity for endometriosis. Reproductive Sciences 30(7), 2040–2059. 10.1007/s43032-023-01167-2. PMC9857924 36662421

[18] Kajimoto T, Mohamed NNI, Badawy SMM, Matovelo SA, Hirase M, Nakamura S, Yoshida D, Okada T, Ijuin T and Nakamura SI (2018) Involvement of gβγ subunits of Gi protein coupled with S1P receptor on multivesicular endosomes in F-actin formation and cargo sorting into exosomes. The Journal of Biological Chemistry 293(1), 245–253. 10.1074/jbc.M117.808733. PMC5766922 29133526

[19] Takuwa Y, Okamoto Y, Yoshioka K and Takuwa N (2008) Sphingosine-1-phosphate signaling and biological activities in the cardiovascular system. Biochimica et Biophysica Acta 1781(9), 483–488. 10.1016/j.bbalip.2008.04.003. 18472021

[20] González-Aretia D, Hernández-Coronado CG, Guzmán A, Medina-Moctezuma ZB, Gutiérrez CG and Rosales-Torres AM (2024) Sphingosine-1-phosphate mediates FSH-induced cell viability but not steroidogenesis in bovine granulosa cells. Theriogenology 213, 90–96. 10.1016/j.theriogenology.2023.10.003. 37820497

[21] Hannun YA and Obeid LM (2008) Principles of bioactive lipid signalling: Lessons from sphingolipids. Nature Reviews. Molecular Cell Biology 9(2), 139–150. 10.1038/nrm2329. 18216770

[22] Colombini M (2017) Ceramide channels and mitochondrial outer membrane permeability. Journal of Bioenergetics and Biomembranes 49(1), 57–64. 10.1007/s10863-016-9646-z. 26801188

[23] Rego A, Costa M, Chaves SR, Matmati N, Pereira H, Sousa MJ, Moradas-Ferreira P, Hannun YA, Costa V and Côrte-Real M (2012) Modulation of mitochondrial outer membrane permeabilization and apoptosis by ceramide metabolism. PLoS One 7(11), e48571. 10.1371/journal.pone.0048571. PMC3511487 23226203

[24] Ganesan V, Perera MN, Colombini D, Datskovskiy D, Chadha K and Colombini M (2010) Ceramide and activated Bax act synergistically to permeabilize the mitochondrial outer membrane. Apoptosis: An International Journal on Programmed Cell Death 15(5), 553–562. 10.1007/s10495-009-0449-0. 20101465

[25] Supakul R and Liangpunsakul S (2011) Alcoholic-induced hepatic steatosis--role of ceramide and protein phosphatase 2A. Translational Research: The Journal of Laboratory and Clinical Medicine 158(2), 77–81. 10.1016/j.trsl.2011.03.005. PMC3137800 21757150

[26] Huo Y, Liu X, Lu C, Li T, Yang Z, Xu F, Chen S, Yin K and Wang L (2025) Ceramide mediates cell-to-cell ER stress transmission by modulating membrane fluidity. The Journal of Cell Biology 224(5), e202405060. 10.1083/jcb.202405060. PMC11938942 40136051

[27] Shibahara H, Ishiguro A, Inoue Y, Koumei S, Kuwayama T and Iwata H (2020) Mechanism of palmitic acid-induced deterioration of in vitro development of porcine oocytes and granulosa cells. Theriogenology 141, 54–61. 10.1016/j.theriogenology.2019.09.006. 31518729

[28] Strub GM, Paillard M, Liang J, Gomez L, Allegood JC, Hait NC, Maceyka M, Price MM, Chen Q, Simpson DC, Kordula T, Milstien S, Lesnefsky EJ and Spiegel S (2011) Sphingosine-1-phosphate produced by sphingosine kinase 2 in mitochondria interacts with prohibitin 2 to regulate complex IV assembly and respiration. FASEB Journal: Official Publication of the Federation of American Societies for Experimental Biology 25(2), 600–612. 10.1096/fj.10-167502. PMC3023391 20959514

[29] Sah RK, Anand S, Dar W, Jain R, Kumari G, Madan E, Saini M, Gupta A, Joshi N, Hada RS, Gupta N, Pati S and Singh S (2023) Host-erythrocytic sphingosine-1-phosphate regulates plasmodium histone deacetylase activity and exhibits epigenetic control over cell death and differentiation. Microbiology Spectrum 11(2), e0276622. 10.1128/spectrum.02766-22. PMC10100792 36744922

[30] Riccio A (2010) New endogenous regulators of class I histone deacetylases. Science Signaling 3(103). 10.1126/scisignal.3103pe1. 20051592

[31] Takuwa Y, Okamoto Y, Yoshioka K and Takuwa N (2012) Sphingosine-1-phosphate signaling in physiology and diseases. BioFactors 38(5), 329–337. 10.1002/biof.1030. 22674845

[32] Wang X, Liu C, Li X, Guo K, Xu S, Zhou J, Wang S, Wu X, Yan G and Cui H (2025) JTE-013 reduces inflammation and improves pulmonary fibrosis by regulating the TRAF2/NF – κB/IGF-1 signaling pathway and macrophage polarization. International Immunopharmacology 167, 115727. 10.1016/j.intimp.2025.115727. 41172817

[33] Kujjo LL and Perez GI (2012) Ceramide and mitochondrial function in aging oocytes: Joggling a new hypothesis and old players. Reproduction 143(1), 1–10. 10.1530/REP-11-0350. 22046054

[34] Kujjo LL, Acton BM, Perkins GA, Ellisman MH, D’Estaing SG, Casper RF, Jurisicova A and Perez GI (2013) Ceramide and its transport protein (CERT) contribute to deterioration of mitochondrial structure and function in aging oocytes. Mechanisms of Ageing and Development 134(1-2), 43–52. 10.1016/j.mad.2012.12.001. 23246342

[35] Perez GI, Jurisicova A, Matikainen T, Moriyama T, Kim MR, Takai Y, Pru JK, Kolesnick RN and Tilly JL (2005) A central role for ceramide in the age-related acceleration of apoptosis in the female germline. FASEB Journal: Official Publication of the Federation of American Societies for Experimental Biology 19(7), 860–862. 10.1096/fj.04-2903fje. 15728664

[36] Zhang X, Zhang L and Xiang W (2025) The impact of mitochondrial dysfunction on ovarian aging. Journal of Translational Medicine 23(1), 211. 10.1186/s12967-025-06223-w. PMC11844166 39980008

[37] Fisher-Wellman KH, Hagen JT, Neufer PD, Kassai M and Cabot MC (2021) On the nature of ceramide-mitochondria interactions – Dissection using comprehensive mitochondrial phenotyping. Cellular Signalling 78, 109838. 10.1016/j.cellsig.2020.109838. PMC7775270 33212155

[38] Ueda N (2015) Ceramide-induced apoptosis in renal tubular cells: A role of mitochondria and sphingosine-1-phoshate. International Journal of Molecular Sciences 16(3), 5076–5124. 10.3390/ijms16035076. PMC4394466 25751724

[39] Kobeissy H, Hage-Sleiman R, Dakdouk Z, Kozhaya L and Dbaibo G (2020) Crosstalk between Noxa, Bcl-2, and ceramide in mediating p53-dependent apoptosis in Molt-4 human T-cell leukemia. Molecular and Cellular Biochemistry 475(1-2), 215–226. 10.1007/s11010-020-03874-9. 32767230

[40] Takashima T, Fujimaru T and Obata Y (2021) Effect of in vitro growth on mouse oocyte competency, mitochondria and transcriptome. Reproduction 162(4), 307–318. 10.1530/REP-21-0209. PMC8494378 34397394

[41] Itami N, Shirasuna K, Kuwayama T and Iwata H (2018) Palmitic acid induces ceramide accumulation, mitochondrial protein hyperacetylation, and mitochondrial dysfunction in porcine oocytes. Biology of Reproduction 98(5), 644–653. 10.1093/biolre/ioy023. 29385411

[42] Kumagai K and Hanada K (2019) Structure, functions and regulation of CERT, a lipid-transfer protein for the delivery of ceramide at the ER-Golgi membrane contact sites. FEBS Letters 593(17), 2366–2377. 10.1002/1873-3468.13511. 31254361

[43] Wang X, Rao RP, Kosakowska-Cholody T, Masood MA, Southon E, Zhang H, Berthet C, Nagashim K, Veenstra TK, Tessarollo L, Acharya U and Acharya JK (2009) Mitochondrial degeneration and not apoptosis is the primary cause of embryonic lethality in ceramide transfer protein mutant mice. The Journal of Cell Biology 184(1), 143–158. 10.1083/jcb.200807176. PMC2615084 19139267

[44] Eliyahu E, Shtraizent N, Shalgi R and Schuchman EH (2012) Construction of conditional acid ceramidase knockout mice and in vivo effects on oocyte development and fertility. Cellular Physiology and Biochemistry: International Journal of Experimental Cellular Physiology, Biochemistry, and Pharmacology 30(3), 735–748. 10.1159/000341453. PMC3741991 22854249

[45] Jain A, Beutel O, Ebell K, Korneev S and Holthuis JCM (2017) Diverting CERT-mediated ceramide transport to mitochondria triggers Bax-dependent apoptosis. Journal of Cell Science 130(2), 360–371. 10.1242/jcs.194191. 27888218

[46] Dapas M and Dunaif A (2022) Deconstructing a syndrome: Genomic insights into PCOS causal mechanisms and classification. Endocrine Reviews 43(6), 927–965. 10.1210/endrev/bnac001. PMC9695127 35026001

[47] Teede HJ, Khomami MB, Morman R, Laven JSE, Joham AE, Costello MF, Patil M, Rees DA, Berry L, Cree MG, Zhao H, Norman RJ, Dokras A, Piltonen T and Global Name Change Consortium (2026) Polyendocrine metabolic ovarian syndrome, the new name for polycystic ovary syndrome: A multistep global consensus process. Lancet 407(10545), 2329–2339. 10.1016/S0140-6736(26)00717-8. 42119588

[48] Brennan KM, Kroener LL, Chazenbalk GD and Dumesic DA (2019) Polycystic ovary syndrome: Impact of lipotoxicity on metabolic and reproductive health. Obstetrical & Gynecological Survey 74(4), 223–231. 10.1097/OGX.0000000000000661. PMC6952118 31344250

[49] Liu L, Yin T-L, Chen Y, Li Y, Yin L, Ding J, Yang J and Feng HL (2019) Follicular dynamics of glycerophospholipid and sphingolipid metabolisms in polycystic ovary syndrome patients. The Journal of Steroid Biochemistry and Molecular Biology 185, 142–149. 10.1016/j.jsbmb.2018.08.008. 30121347

[50] Cordeiro FB, Cataldi TR, do Vale Teixeira da Costa L, de Lima CB, Stevanato J, Zylbersztejn DS, Ferreira CR, Eberlin MN, Cedenho AP and Turco EGL (2015) Follicular fluid lipid fingerprinting from women with PCOS and hyper response during IVF treatment. Journal of Assisted Reproduction and Genetics 32(1), 45–54. 10.1007/s10815-014-0375-0. PMC4294864 25374394

[51] Li S, Chu Q, Ma J, Sun Y, Tao T, Huang R, Liao Y, Yue J, Zheng J, Wang L, Xue X, Zhu M, Kang X, Yin H and Liu W (2017) Discovery of novel lipid profiles in PCOS: Do insulin and androgen oppositely regulate bioactive lipid production? The Journal of Clinical Endocrinology and Metabolism 102(3), 810–821. 10.1210/jc.2016-2692. PMC5477809 27886515

[52] Shi W, Lin H, Di W, He C and Shen Y (2025) Granulosa cell RNA-Seq insights into senescence and sphingolipid metabolism disorder in PCOS: aspirin as a potential therapeutic drug. Reproductive biology and endocrinology 23(1), 61. 10.1186/s12958-025-01396-x. PMC12032776 40287692

[53] Ding Y, Jiang Y, Zhu M, Zhu Q, He Y, Lu Y, Wang Y, Qi J, Feng Y, Huang R, Yin H, Li S and Sun Y (2022) Follicular fluid lipidomic profiling reveals potential biomarkers of polycystic ovary syndrome: A pilot study. Frontiers in Endocrinology 13, 960274. 10.3389/fendo.2022.960274. PMC9513192 36176459

[54] Yao L, Wang Q, Zhang R, Wang X, Liu Y, Di F, Song L and Xu S (2021) Brown adipose transplantation improves polycystic ovary syndrome-involved metabolome remodeling. Frontiers in Endocrinology 12, 747944. 10.3389/fendo.2021.747944. PMC8667175 34912296

[55] Pradas I, Rovira-Llopis S, Naudí A, Bañuls C, Rocha M, Hernandez-Mijares A, Pamplona R, Victor VM and Jové M (2019) Metformin induces lipid changes on sphingolipid species and oxidized lipids in polycystic ovary syndrome women. Scientific Reports 9(1), 16033. 10.1038/s41598-019-52263-w. PMC6831788 31690730

[56] Shi Y, Zhao H, Shi Y, Cao Y, Yang D, Li Z, Zhang B, Liang X, Li T, Chen J, Shen J, Zhao J, You L, Gao X, Zhu D, Zhao X, Yan Y, Qin Y, Li W, Yan J, Wang Q, Zhao J, Geng L, Ma J, Zhao Y, He G, Zhang A, Zou S, Yang A, Liu J, Li W, Li B, Wan C, Qin Y, Shi J, Yang J, Jiang H, Xu JE, Qi X, Sun Y, Zhang Y, Hao C, Ju X, Zhao D, Ren CE, Li X, Zhang W, Zhang Y, Zhang J, Wu D, Zhang C, He L and Chen ZJ (2012) Genome-wide association study identifies eight new risk loci for polycystic ovary syndrome. Nature Genetics 44(9), 1020–1025. 10.1038/ng.2384. 22885925

[57] Knapp P, Chomicz K, Świderska M, Chabowski A and Jach R (2019) Unique roles of sphingolipids in selected malignant and nonmalignant lesions of female reproductive system. BioMed Research International 2019, 1–7. 10.1155/2019/4376583. PMC6521305 31187044

[58] Sutphen R, Xu Y, Wilbanks GD, Fiorica J, Grendys Jr EC, LaPolla JP, Arango H, Hoffman MS, Martino M, Wakeley K, Griffin D, Blanco RW, Cantor AB, Xiao YJ and Krischer JP (2004) Lysophospholipids are potential biomarkers of ovarian cancer. Cancer Epidemiology, Biomarkers & Prevention 13(7), 1185–1191. 10.1158/1055-9965.1185.13.7. 15247129

[59] Li J, Zhi X, Lin Q, Sun Y, Sun Y, Abudousalamu Z, Xue M, Zheng C, Li X, Yao L and Chen M (2026) MEOX1 Coordinates Autocrine-Paracrine Programs via SPHK1/S1P to Promote Lymph Node Metastasis in Ovarian Cancer. Advanced Science 13(43), e23574. 10.1002/advs.202523574. PMC13336135 42107070

[60] Tao Y-P, Zhu H-Y, Shi Q-Y, Wang CX, Hua YX, Hu HY, Zhou QY, Zhou ZL, Sun Y, Wang XM, Wang Y, Zhang YL, Guo YJ, Wang ZY, Che X, Xu CW, Zhang XC, Heger M, Tao SP, Zheng X, Xu Y, Ao L, Liu AJ, Liu SB, Cheng SQ and Pan WW (2023) S1PR1 regulates ovarian cancer cell senescence through the PDK1-LATS1/2-YAP pathway. Oncogene 42(47), 3491–3502. 10.1038/s41388-023-02853-w. PMC10656284 37828220

[61] Dai L, Wang C, Song K, Wang W and di W (2021) Activation of SphK1 by adipocytes mediates epithelial ovarian cancer cell proliferation. Journal of Ovarian Research 14(1), 62. 10.1186/s13048-021-00815-y. PMC8088075 33931106

[62] Hernández-Coronado CG, Guzmán A, Rodríguez A, Mondragón JA, Romano MC, Gutiérrez CG, and Rosales-Torres AM (2016) Sphingosine-1-phosphate, regulated by FSH and VEGF, stimulates granulosa cell proliferation. General and Comparative Endocrinology 236, 1–8. 10.1016/j.ygcen.2016.06.029. 27342378

[63] Visentin B, Vekich JA, Sibbald BJ, Cavalli AL, Moreno KM, Matteo RG, Garland WA, Lu Y, Yu S, Hall HS, Kundra V, Mills GB and Sabbadini RA (2006) Validation of an anti-sphingosine-1-phosphate antibody as a potential therapeutic in reducing growth, invasion, and angiogenesis in multiple tumor lineages. Cancer Cell 9(3), 225–238. 10.1016/j.ccr.2006.02.023. 16530706

[64] Pyne NJ, Tonelli F, Lim KG, Long JS, Edwards J and Pyne S (2012) Sphingosine 1-phosphate signalling in cancer. Biochemical Society Transactions 40(1), 94–100. 10.1042/BST20110602. 22260672

[65] Sawada M, Nakashima S, Banno Y, Yamakawa H, Takenaka K, Shinoda J, Nishimura Y, Sakai N and Nozawa Y (2000) Influence of Bax or Bcl-2 overexpression on the ceramide-dependent apoptotic pathway in glioma cells. Oncogene 19(31), 3508–3520. 10.1038/sj.onc.1203699 10918609

[66] Hernández-Coronado CG, Guzmán A, Espinosa-Cervantes R, Romano MC, Verde-Calvo JR and Rosales-Torres AM (2015) Sphingosine-1-phosphate and ceramide are associated with health and atresia of bovine ovarian antral follicles. Animal: An International Journal of Animal Bioscience 9(2), 308–312. 10.1017/S1751731114002341. 25245232

[67] Van Brocklyn JR and Williams JB (2012) The control of the balance between ceramide and sphingosine-1-phosphate by sphingosine kinase: Oxidative stress and the seesaw of cell survival and death. Comparative Biochemistry and Physiology. Part B, Biochemistry & Molecular Biology 163(1), 26–36. 10.1016/j.cbpb.2012.05.006. 22613819

[68] Touraine P, Chabbert-Buffet N, Plu-Bureau G, Duranteau L, Sinclair AH and Tucker EJ (2024) Premature ovarian insufficiency. Nature Reviews. Disease Primers 10(1), 63. 10.1038/s41572-024-00547-5. 39266563

[69] Ogretmen B (2018) Sphingolipid metabolism in cancer signalling and therapy. Nature Reviews. Cancer 18(1), 33–50. 10.1038/nrc.2017.96. PMC5818153 29147025

[70] Giaccari C, Antonouli S, Anifandis G, Cecconi S and di Nisio V (2024) An update on physiopathological roles of Akt in the ReprodAKTive mammalian ovary. Life 14(6), 722. 10.3390/life14060722. PMC11204812 38929705

[71] Lee S, Kang H-G, Jeong P-S, Kim MJ, Park SH, Song BS, Sim BW and Kim SU (2021) Heat stress impairs oocyte maturation through ceramide-mediated apoptosis in pigs. The Science of the Total Environment 755(Pt 1), 144144. 10.1016/j.scitotenv.2020.144144. 33288257

[72] Ueda N (2022) A rheostat of ceramide and sphingosine-1-phosphate as a determinant of oxidative stress-mediated kidney injury. International Journal of Molecular Sciences 23(7), 4010. 10.3390/ijms23074010. PMC9000186 35409370

[73] Practice Committee of the American Society for Reproductive Medicine. Electronic address: ASRM@asrm.org and Practice Committee of the American Society for Reproductive Medicine (2016) Prevention and treatment of moderate and severe ovarian hyperstimulation syndrome: A guideline. Fertility and Sterility 106, 1634–1647. 10.1016/j.fertnstert.2016.08.048 27678032

[74] Ata B and Humaidan P (2025) Ovarian hyperstimulation syndrome – A complication of the past. Reproductive Biomedicine Online 50(4), 104792. 10.1016/j.rbmo.2024.104792. 40287203

[75] Scotti L, Di Pietro M, Pascuali N, Irusta G, de Zúñiga I, Gomez Peña M, Pomilio C, Saravia F, Tesone M, Abramovich D and Parborell F (2016) Sphingosine-1-phosphate restores endothelial barrier integrity in ovarian hyperstimulation syndrome. Molecular Human Reproduction 22(12), 852–866. 10.1093/molehr/gaw065. 27645281

[76] Di Pietro M, Pascuali N, Scotti L, Irusta G, Bas D, May M, Tesone M, Abramovich D and Parborell F (2017) In vivo intrabursal administration of bioactive lipid sphingosine-1-phosphate enhances vascular integrity in a rat model of ovarian hyperstimulation syndrome. Molecular Human Reproduction 23(6), 417–427. 10.1093/molehr/gax021. 28379469

[77] Smolarz B, Szyłło K and Romanowicz H (2021) Endometriosis: epidemiology, classification, pathogenesis, treatment and genetics (review of literature). International Journal of Molecular Sciences 22(19), 10554. 10.3390/ijms221910554. PMC8508982 34638893

[78] Lin N, Zeleznik OA, Vitonis AF, Laliberte A, Shafrir AL, Avila-Pacheco J, Clish C, Terry KL, Missmer SA and Sasamoto N (2025) Plasma metabolites associated with endometriosis in adolescents and young adults. Human Reproduction 40(5), 843–854. 10.1093/humrep/deaf040. PMC12046077 40107296

[79] Lee YH, Tan CW, Venkatratnam A, Tan CS, Cui L, Loh SF, Griffith L, Tannenbaum SR and Chan JKY (2014) Dysregulated sphingolipid metabolism in endometriosis. The Journal of Clinical Endocrinology and Metabolism 99(10), E1913–1921. 10.1210/jc.2014-1340. PMC5393497 24960545

[80] Vouk K, Hevir N, Ribić-Pucelj M, Haarpaintner G, Scherb H, Osredkar J, Möller G, Prehn C, Lanišnik Rižner T and Adamski J (2012) Discovery of phosphatidylcholines and sphingomyelins as biomarkers for ovarian endometriosis. Human Reproduction 27(10), 2955–2965. 10.1093/humrep/des152. 22859507

[81] Prisinzano M, Bernacchioni C, Seidita I, Rossi M, Raeispour M, Cencetti F, Vannuccini S, Fambrini M, Petraglia F, Bruni P and Donati C (2024) Sphingosine 1-phosphate signaling axis mediates neuropeptide S-induced invasive phenotype of endometriotic cells. The FEBS Journal 291(8), 1744–1758. 10.1111/febs.17071. 38287231

[82] Weigert A, Johann AM, von Knethen A, Schmidt H, Geisslinger G and Brüne B (2006) Apoptotic cells promote macrophage survival by releasing the antiapoptotic mediator sphingosine-1-phosphate. Blood 108, 1635–1642. 10.1182/blood-2006-04-014852 IF: 23.9 Q1 B1. 16690965

[83] Ono Y, Kawakita T, Yoshino O, Sato E, Kano K, Ohba M, Okuno T, Ito M, Koga K, Honda M, Furue A, Hiraoka T, Wada S, Iwasa T, Yokomizo T, Aoki J, Maeda N, Unno N, Osuga Y and Hirata S (2021) Sphingosine 1-phosphate (S1P) in the peritoneal fluid skews M2 macrophage and contributes to the development of endometriosis. Biomedicine 9(11), 1519. 10.3390/biomedicines9111519. PMC8614877 34829748

[84] Yoshino O, Yamada-Nomoto K, Kano K, Ono Y, Kobayashi M, Ito M, Yoneda S, Nakashima A, Shima T, Onda T, Osuga Y, Aoki J and Saito S (2019) Sphingosine 1 phosphate (S1P) increased IL-6 expression and cell growth in endometriotic cells. Reproductive Sciences 26(11), 1460–1467. 10.1177/1933719119828112. 30782093

[85] An M, Li D, Yuan M, Li Q, Zhang L and Wang G (2017) Interaction of macrophages and endometrial cells induces epithelial-mesenchymal transition-like processes in adenomyosis. Biology of Reproduction 96(1), 46–57. 10.1095/biolreprod.116.144071. 28395325

[86] Zhang Q, Duan J, Olson M, Fazleabas A and Guo SW (2016) Cellular changes consistent with epithelial-mesenchymal transition and fibroblast-to-myofibroblast transdifferentiation in the progression of experimental endometriosis in baboons. Reproductive Sciences 23(10), 1409–1421. 10.1177/1933719116641763. PMC5933178 27076446

[87] Quarta S, Camprubí-Robles M, Schweigreiter R, Matusica D, Haberberger RV, Proia RL, Bandtlow CE, Ferrer-Montiel A and Kress M (2017) Sphingosine-1-phosphate and the S1P3 receptor initiate neuronal retraction via RhoA/ROCK associated with CRMP2 phosphorylation. Frontiers in Molecular Neuroscience 10, 317. 10.3389/fnmol.2017.00317. PMC5641356 29066950

[88] Gavard J and Gutkind JS (2006) VEGF controls endothelial-cell permeability by promoting the beta-arrestin-dependent endocytosis of VE-cadherin. Nature Cell Biology 8(11), 1223–1234. 10.1038/ncb1486. 17060906

[89] Semenza GL (2003) Angiogenesis ischemic and neoplastic disorders. Annual Review of Medicine 54(1), 17–28. 10.1146/annurev.med.54.101601.152418. 12359828

[90] Vannuzzi V, Bernacchioni C, Muccilli A, Castiglione F, Nozzoli F, Vannuccini S, Capezzuoli T, Ceccaroni M, Bruni P, Donati C and Petraglia F (2022) Sphingosine 1-phosphate pathway is dysregulated in adenomyosis. Reproductive Biomedicine Online 45(1), 15–18. 10.1016/j.rbmo.2022.03.026. 35562234

[91] Bernacchioni C, Capezzuoli T, Vannuzzi V, Malentacchi F, Castiglione F, Cencetti F, Ceccaroni M, Donati C, Bruni P and Petraglia F (2021) Sphingosine 1-phosphate receptors are dysregulated in endometriosis: Possible implication in transforming growth factor β-induced fibrosis. Fertility and Sterility 115(2), 501–511. 10.1016/j.fertnstert.2020.08.012. 32907751

[92] Vannuccini S, Gabbrielli S, Castiglione F, Nardi E, Rossi M, Orlandi G, Wu E, Bertoli F, Miele V, Donati C and Petraglia F (2024) Imaging and molecular features of adenomyosis after menopause. Maturitas 185, 107996. 10.1016/j.maturitas.2024.107996. 38657487

[93] Chrobak A, Sieradzka U, Sozański R, Chełmońska-Soyta A, Gabryś M and Jerzak M (2009) Ectopic and eutopic stromal endometriotic cells have a damaged ceramide signaling pathway to apoptosis. Fertility and Sterility 92(6), 1834–1843. 10.1016/j.fertnstert.2008.09.035. 19019359

[94] Rossi M, Seidita I, Prisinzano M, Raeispour M, Romeo L, Sorbi F, Fambrini M, Ciarmela P, Petraglia F, Bernacchioni C and Donati C (2025) Sphingosine 1-phosphate acts as proliferative and fibrotic cue in leiomyoma cells. F&S Science 6(1), 99–106. 10.1016/j.xfss.2024.11.003. 39643001

[95] Bernacchioni C, Ciarmela P, Vannuzzi V, Greco S, Vannuccini S, Malentacchi F, Pellegrino P, Capezzuoli T, Sorbi F, Cencetti F, Bruni P, Donati C and Petraglia F (2021) Sphingosine 1-phosphate signaling in uterine fibroids: Implication in activin a pro-fibrotic effect. Fertility and Sterility 115(6), 1576–1585. 10.1016/j.fertnstert.2020.12.022. 33500141

[96] Islam MS, Akhtar MM and Segars JH (2021) S1P and activin a induce fibrotic phenotype in uterine fibroids: A promising target for antifibrotic therapy. Fertility and Sterility 115(6), 1451–1452. 10.1016/j.fertnstert.2021.03.021. 33863554

[97] Fakhr Y, Brindley DN and Hemmings DG (2021) Physiological and pathological functions of sphingolipids in pregnancy. Cellular Signalling 85, 110041. 10.1016/j.cellsig.2021.110041. 33991614

[98] Ye Q, Zeng X, Cai S, Qiao S and Zeng X (2021) Mechanisms of lipid metabolism in uterine receptivity and embryo development. Trends in Endocrinology and Metabolism: TEM 32(12), 1015–1030. 10.1016/j.tem.2021.09.002. 34625374

[99] Kaneko-Tarui T, Zhang L, Austin KJ, Henkes LE, Johnson J, Hansen TR and Pru JK (2007) Maternal and embryonic control of uterine sphingolipid-metabolizing enzymes during murine embryo implantation. Biology of Reproduction 77(4), 658–665. 10.1095/biolreprod.107.061044. 17582011

[100] Ding N-Z, Qi Q-R, Gu X-W, Zuo RJ, Liu J and Yang ZM (2018) *De novo* synthesis of sphingolipids is essential for decidualization in mice. Theriogenology 106, 227–236. 10.1016/j.theriogenology.2017.09.036. 29096270

[101] Wang Y, Chen X, You G, Yu S, Chen C, Lian R, Diao L, Li Y and Yin T (2026) CEBPD-mediated SGPP2 upregulation via PERK/ER stress in endothelial cells disrupts S1P homeostasis and impairs angiogenesis in chronic endometritis. Journal of Translational Medicine 24(1), 98. 10.1186/s12967-025-07558-0. PMC12822190 41408309

[102] Labus JS, Mayer EA, Tillisch K, Aagaard KM, Stains J, Broniowska K, van Remortel C, Tun G and Rapkin A (2021) Dysregulation in sphingolipid signaling pathways is associated with symptoms and functional connectivity of pain processing brain regions in provoked vestibulodynia. The Journal of Pain 22(12), 1586–1605. 10.1016/j.jpain.2021.04.017. PMC10460622 34029688

[103] Stanisz AM and Stanisz JA (2000) Nerve growth factor and neuroimmune interactions in inflammatory diseases. Annals of the New York Academy of Sciences 917(1), 268–272. 10.1111/j.1749-6632.2000.tb05392.x. 11268353

[104] Zhang X, Zhou Y, Wang C, Ren J, Wang Y, Liu P, Feng W, Li X, Qi M, Yang Y, Zhu C, Wang F, Ma Y, Tang Z and Yu G (2025) S1P/S1PRs-TRPV4 axis is a novel therapeutic target for persistent pain and itch in chronic dermatitis. British Journal of Pharmacology 182(5), 1223–1235. 10.1111/bph.17393. 39581852

[105] Gonzalez PA, Simcox J, Raff H, Wade G, von Bank H, Weisman S and Hainsworth K (2022) Lipid signatures of chronic pain in female adolescents with and without obesity. Lipids in Health and Disease 21(1), 80. 10.1186/s12944-022-01690-2. PMC9426222 36042489

[106] Timur B, Aldemir O, İnan N, Kaplanoğlu İ and Dilbaz S (2022) Clinical significance of serum and follicular fluid ceramide levels in women with low ovarian reserve. Turkish Journal of Obstetrics and Gynecology 19(3), 207–214. 10.4274/tjod.galenos.2022.05760. PMC9511935 36149261

[107] Batushansky A, Zacharia A, Shehadeh A, Bruck-Haimson R, Saidemberg D, Kogan NM, Thomas Mannully C, Herzberg S, Ben-Meir A and Moussaieff A (2020) A shift in glycerolipid metabolism defines the follicular fluid of IVF patients with unexplained infertility. Biomolecules 10(8), 1135. 10.3390/biom10081135. PMC7465802 32752038

[108] Knapp P, Bodnar L, Błachnio-Zabielska A, Świderska M and Chabowski A (2017) Plasma and ovarian tissue sphingolipids profiling in patients with advanced ovarian cancer. Gynecologic Oncology 147(1), 139–144. 10.1016/j.ygyno.2017.07.143. 28800942

[109] Pascuali N, Scotti L, Di Pietro M, Oubiña G, Bas D, May M, Muñoz AG, Cuasnicú PS, Cohen DJ, Tesone M, Abramovich D and Parborell F (2018) Ceramide-1-phosphate has protective properties against cyclophosphamide-induced ovarian damage in a mice model of premature ovarian failure. Human Reproduction 33(5), 844–859. 10.1093/humrep/dey045. 29534229

